# Smartphone‐Assisted Wireless Ultrasensitive Nitrite Detection in Food Samples via Hierarchical MXene/NiCoMn‐LDH/Sulfide Heterostructure on Flexible Laser‐Induced Graphene Electrode

**DOI:** 10.1002/smll.202510411

**Published:** 2025-11-12

**Authors:** Kugalur Shanmugam Ranjith, Ali Mohammadi, A. T. Ezhil Vilian, Yonghyeon Park, Ganji Seeta Rama Raju, Yun Suk Huh, Young‐Kyu Han

**Affiliations:** ^1^ Department of Energy and Material Engineering Dongguk University‐Seoul Seoul 04620 Republic of Korea; ^2^ Department of Biological Sciences and Bioengineering Nano Bio High‐Tech Materials Research Center Inha University Incheon 22212 Republic of Korea

**Keywords:** electrochemical detection, flexible electrode, food safety, MXene, nitrite sensor

## Abstract

Designing a smartphone in‐built, wireless, ultrasensitive hybrid heterostructure‐based sensor is challenging, but it is potentially crucial for achieving efficient electrochemical responses related to real‐time food safety monitoring. Herein, the pioneering design of a few‐layered MXene‐tagged NiCoMn‐layered double hydroxide (LDH)/amorphous sulfide (MXe/NiCoMn‐LDH/S) hollow spheres were fabricated and tagged onto a laser‐induced graphene (LIG) electrode for smartphone‐based electrochemical detection of nitrite (NO_2_^−^). Benefiting from the unique structural and compositional advantages of MXe/NiCoMn‐LDH/S integrated on a flexible LIG electrode platform, the sensor achieved a linear detection range from 0.90 to 25 µM, and a low detection limit of 0.21 µM, along with a high sensitivity of 11.88 µA µM^−1^ cm^−2^. Additionally, using the LSV, the sensor demonstrated a wider linear range from 10 to 860 µM with a detection limit of 7.38 µM and sensitivity of 1.15 µA µM^−1^ cm^−2^. It also demonstrated strong anti‐interference capability against various organic and inorganic substances, along with excellent reproducibility, repeatability, and reusability, as well as outstanding stability over 30 days. The proposed point‐of‐care electrochemical system, featuring a portable and affordable LIG electrode design, along with compact smartphone integrity, highlights its advantages over traditional benchtop potentiostats in real‐time water quality monitoring in rural and low‐resource areas.

## Graphical abstract

Surface‐enriched MXene‐tagged NiCoMn‐layered double hydroxide (LDH)/amorphous sulfide (MXe/NiCoMn‐LDH/S) hollow spheres tagged laser‐induced graphene (LIG) electrode for smartphone‐based electrochemical detection of nitrite (NO_2_^−^), real‐time water quality monitoring.

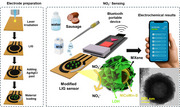

## 1 Introduction

The development of efficient and resourceful catalysts integrated within a portable, smartphone‐based electrochemical sensing platform presents a formidable challenge in the realm of sustainable environmental technologies.^[^ ^1^ ^]^ Such systems are pivotal for the real‐time detection of pollutants in aqueous environments, particularly in applications related to food safety and public health monitoring. The rapid development and expansion of agricultural and industrial activities have resulted in the discharge of a wide range of contaminants into aquatic environments, eliciting profound concerns regarding human health and ecological stability.^[^ ^2^ ^]^ Among these pollutants, nitrite ions (NO_2_^−^) represent a significant class of inorganic nitrogenous contaminants that pose a serious risk to environmental and human health due to their high toxicity and bio‐accumulative nature. Widely used across industries such as agriculture, food processing, chemical manufacturing, and pharmaceuticals, excessive exposure to nitrites—particularly through contaminated water sources can lead to severe health effects, including acute poisoning, neurological damage, and increased cancer risk.^[^ ^3^, ^4^ ^]^ The World Health Organization (WHO) has given the guideline that the maximum regulated level of nitrite in ground water supplies is ≈3 mg L^−1^ (65.2 µM), and the European Food Safety Authority (EFSA) has issued an advisory appraisal for nitrite and nitrate with the maximum permitted level of NO_2_^−^ at 180‒500 mg kg^−1^ range as an additive in animal feed.^[^ ^5^ ^]^ In groundwater, concentrations of NO_2_^−^ are lower than those of nitrate (NO_3_^−^) and ammonia; yet, their environmental toxicity is significantly high, which initiates the development of a rapid, simple, and effective NO_2_^−^ sensor for food safety.^[^ ^6^ ^]^ Various methods are commonly used to determine NO_2_^−^, including techniques such as chromatography,^[^ ^7^ ^]^ spectrophotometry,^[^ ^8^, ^9^ ^]^ chemiluminescence,^[^ ^10^ ^]^ spectrophotometric,^[^ ^11^ ^]^ and electrochemical^[^ ^12^, ^13^ ^]^ methods. While several approaches exist for the accurate detection of NO_2_^−^, they are constrained by restrictions such as the need for costly equipment, intricate detection procedures, and unsuitability for on‐site analysis.^[^ ^5^ ^]^ Among these, electrochemical sensors have excellent selectivity, sensitivity, dependability, and quick reaction times, making them ideal for both quantitative and qualitative NO_2_^–^ detection.^[^ ^4^, ^14^ ^]^ To promote the on‐site detection capabilities of traditional electrochemical analysis, portable electrochemical technology integrated with smartphones offers significant advantages in the fields of point‐of‐care (POC) testing and mobile diagnostics. With the simplified electronic design, reduced volume, and lower cost of the system, the POC‐based devices, as a handheld detector, can perform electrochemical measurements and transmit the results to a smartphone via Bluetooth.^[^ ^15^ ^]^

In the design of electrocatalytic materials, considerations of sustainability and the practical applicability of the electrode for commercial use are paramount. Incorporating graphene‐based systems into the modified electrode architecture can significantly enhance electrocatalytic performance, owing to graphene's exceptional electrical conductivity, large surface area, and chemical stability, thereby improving the overall electrochemical response.^[^ ^16^ ^]^ The fabrication of laser‐induced graphene (LIG) as an electrode substrate, which is obtained through thermal deformation of polymer through laser etching, dramatically improves the electrode's excellent conductivity. It also serves as a new kind of flexible and paper‐based electrode assembly, which lessens the electrode's fabrication cost, makes it more portable, and opens up new possibilities for real‐time, on‐site electrochemical detection.^[^ ^17^ ^]^ However, unmodified LIG has a limitation on electrochemical sensing due to its intrinsic structural and chemical properties, as well as potential alterations introduced during the modification process. These factors can affect the material's surface reactivity, electron transfer kinetics, and overall sensing performance. To enhance functionality, LIGs can be hybridized with semiconductor, metal‐organic frameworks, and metal nanostructures, which improve sensitivity, selectivity, and stability by increasing conductivity and active surface sites on the electrode surface.^[^ ^18^, ^19^ ^]^ Despite the widespread use of high‐cost noble metal‐based electrodes in electrochemical sensors, the advancement of nanoengineered materials with enhanced electrochemical performance is crucial for promoting the commercial viability and scalability of NO_2_^−^ sensing technologies. By strategically designing cost‐effective nanomaterials with superior catalytic and conductive properties, it is possible to overcome economic barriers while maintaining high sensitivity and selectivity in sensor performance. Designing single‐transition‐metal‐sensitive electrodes is primarily susceptible to nitrate interference due to the similarity between NO_2_^−^ and NO_3_^−^, which compromises the electrode's selectivity and sensitivity.^[^ ^20^ ^]^ In previous reports, constructing the hybrid systems with transition metal‐based composite heterostructures has drawn significant attention to promoting highly efficient electrochemical sensitivity and stability.^[^ ^21^ ^]^ For example, Liu et al.^[^ ^22^ ^]^ enhanced nitrite sensing by designing Au/NiO/Rh heterostructures, achieving a low detection limit of 0.3 µM, a wide linear range (1 µM–1 mM), and strong anti‐interference capability. Similarly, Zhe et al.^[^ ^23^ ^]^ developed a non‐planar FeCo‐based MOF heterostructure, where the abundant Fe–O–Co–O–Fe interfaces improved surface reaction kinetics, resulting in excellent electrochemical sensing performance.

Engineering transition metal sulfides with enriched defect sites enhances electrocatalytic activity by regulating the electronic structure and electrical conductivity, outperforming their metal oxide counterparts.^[^ ^24^ ^]^ The implementation of amorphous nanostructures with a random arrangement of atoms enhances electrocatalytic performance by increasing reactive sites, reducing structural strain, and facilitating electrolyte interaction.^[^ ^25^, ^26^ ^]^ Compared to monometallic and bimetallic sulfides, trimetallic or mixed sulfides exhibit superior redox activity, accelerated electron transfer, and enriched faradaic reactions. Leveraging these structural advantages, electrode materials such as NiCoFe‐LDH/sulfides,^[^ ^27^ ^]^ MoS_2_/MnS/SnS,^[^ ^28^ ^]^ CoNiZnS/C,^[^ ^29^ ^]^ Mn–Ni–Co sulfides,^[^ ^30^ ^]^ and NiCoMn sulfides^[^ ^31^ ^]^ have been designed with improved electrochemical properties. However, designing mixed‐metal or trimetallic transition metal electrocatalysts faces challenges such as limited activity, poor conductivity, inadequate exposure of electrocatalytic sites, uncontrolled migration, and aggregation.^[^ ^25^, ^31^, ^32^ ^]^ To resolve these issues, strategies like architecture hybridization, heteroatom doping, and heterostructure interface engineering have been employed to improve the electrochemical performance of these materials significantly.

MXene, a 2D transition metal carbide nanomaterial, boasts exceptional conductivity and rich surface functionalities, providing abundant active sites for mixed metal composites.^[^ ^33^ ^]^ However, like other 2D materials, MXene suffers from restacking and aggregation issues, which limit its specific surface area and hinder its ionic diffusion mobility. Simultaneously improving structural and catalytic stability may be accomplished by intercalating multilayer MXene onto structurally designed metal composites, which would also promote high intrinsic activity and expose many active sites.^[^ ^34^ ^]^ A practical approach to improving electrochemical performance might be to design a hybridized platform with a distinct configuration that provides benefits over dual manipulation of metal composites and MXene layers. This functionality promotes the benefits of developing a hybrid electrode for electrochemical technology. Integrating layered MXene with structurally engineered metal composites not only enhances intrinsic activity and exposes multiple active sites but also improves structural and catalytic stability, addressing key performance challenges.^[^ ^35^, ^36^ ^]^

Building on the concepts discussed, a portable electrode assembly was developed featuring hybridized few‐layered MXene‐wrapped hierarchical trimetallic NiCoMn‐layered double hydroxide (LDH)/amorphous sulfide (MXe/NiCoMn‐LDH/S) hollow spheres integrated onto flexible LIG‐based electrodes. This platform, coupled with smartphone‐based wireless connectivity, enables sensitive and real‐time electrochemical detection of (NO_2_^−^), facilitating efficient water quality monitoring. In this work, we present a unique 2D‐3D hybrid heterostructure comprising nanoengineered MXe/NiCoMn‐LDH/S hollow spheres, synthesized via an anion exchange process followed by functionalization of MXene. The resulting heterostructure, formed through strong interfacial bonding between the MXene‐based shell and amorphous mixed‐metal sulfides, effectively inherits and integrates their favorable physicochemical properties. This configuration demonstrates superior structural versatility and a pronounced synergistic effect, which collectively optimize the electron configuration and significantly enhance the density and accessibility of electrocatalytically active sites. Considering the synergistic effects and the abundance of electrocatalytically active surface centers at the hybrid heterogeneous interface of the catalytic spheres, the sensor exhibited an improved linear detection range from 0.90 to 25 µM and a low detection limit of 0.21 µM, with reliable real‐time sample analysis. The incorporation of a thin, decorated MXene layer significantly enhanced the structural integrity and electrocatalytic durability of the NiCoMn‐LDH/S hollow spheres by facilitating efficient electron transport, thereby improving overall electrochemical performance. Key analytical merits such as reproducibility, repeatability, and reusability were systematically evaluated. The resulting portable electrochemical system demonstrated strong potential for POC applications, particularly in detecting nitrite in real wastewater samples.

## 2 Results and Discussion

### 2.1 Structural Characterization

**Scheme 1:**
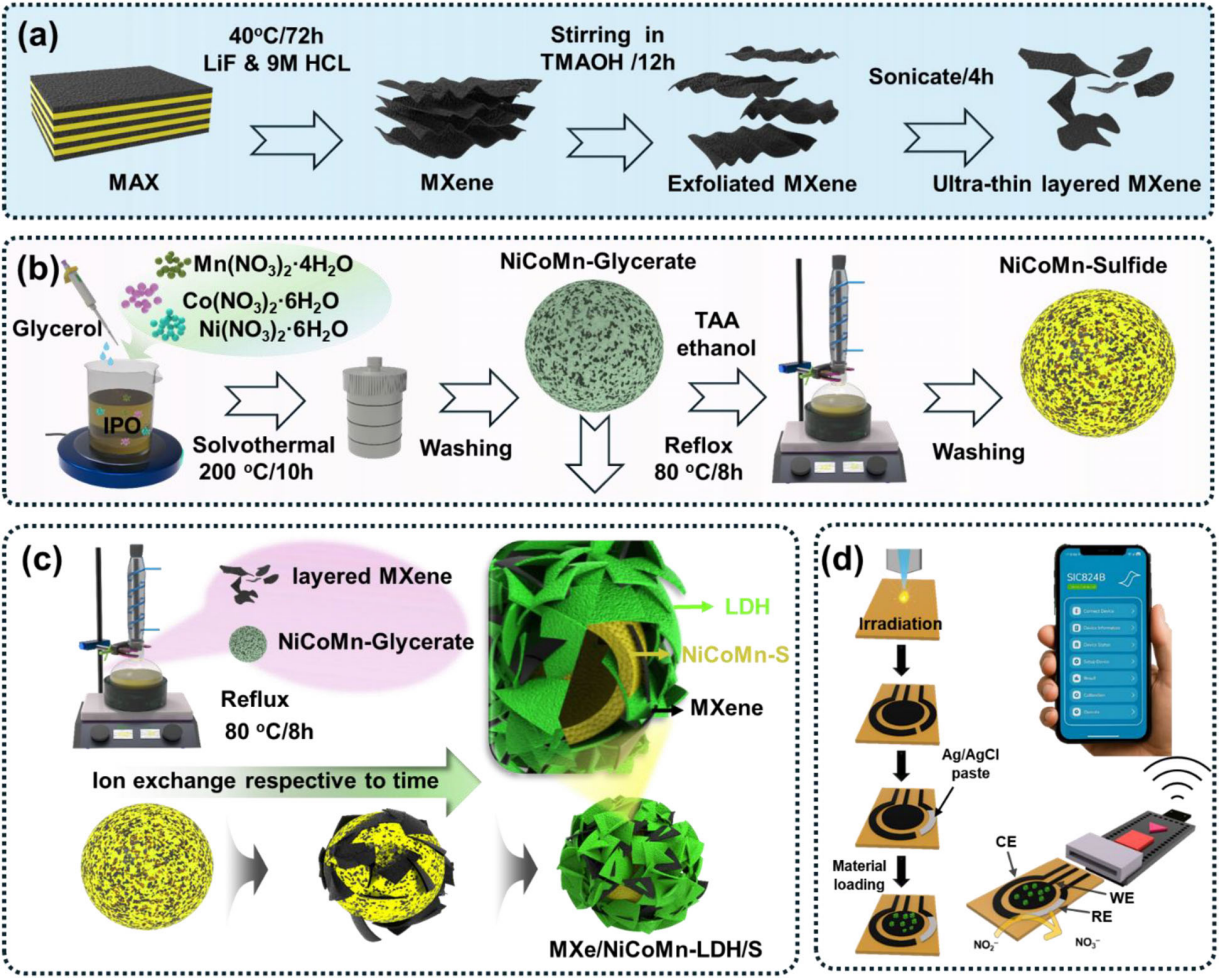
a–c) Schematic diagram illustrating the preparation of hybridized MXe/NiCoMn‐LDH/S hollow spheres and d) the electrode integrity on the LIG substrate and the POC testing system.

The fabrication process of NiCoMn‐glycerate solid spheres and their subsequent transformation into NiCoMn‐sulfide wrapped with MXene, resulting in MXe/NiCoMn‐LDH/S hollow spheres, is illustrated in **Scheme** **1**. First, the few‐layered MXene was prepared through the selective removal of Al by acid etching of the MAX phase, followed by an intercalation and exfoliation process (Scheme 1a). Second, through the solvothermal process, a uniform solid mixed metal glycerate was prepared, which then transformed into hollow NiCoMn‐sulfide through sulfidation treatment in a thioacetamide/ethanol solution (Scheme 1b). In this case, the released S^2^, with the inward diffusion and outward diffusion of mixed metal ions at elevated temperatures, favors the formation of the NiCoMn‐sulfide shell. As the progress continues, the secondary layer of NiCoMn‐sulfide shell wall is prepared on the primary nucleus, and the favorable ion exchange process results in the amorphous NiCoMn‐sulfide hollow spheres. Simultaneously, adding the well‐dispersed MXene during the ion exchange process, wrapped on the shell wall through the electrostatic functionality, and obtained the heterostructure MXene‐tagged NiCoMn‐S hollow spheres. Specifically tagging the MXene onto the NiCoMn‐glycerate during the ion exchange process resulted in the favorable growth of LDH on the NiCoMn‐sulfide, leading to the heterostructure functionality of MXene/NiCoMn‐LDH/S as a hierarchical hollow sphere (Scheme 1c). The fabricated heterostructures were drop‐cast onto the LIG electrode pattern derived from a polyamine substrate, and the electrochemical performance of the flexible electrodes was monitored toward nitrite in real food samples. By integrating the POC testing system with smartphone integrity (Scheme 1d), the Lab‐to‐field electrochemical analysis was conducted to overcome the limitations of traditional bench‐top‐based electrochemical sensor analysis. The detailed preparation process and characterization details are provided in the Experimental Section of the Supporting Information.

**Figure 1:**
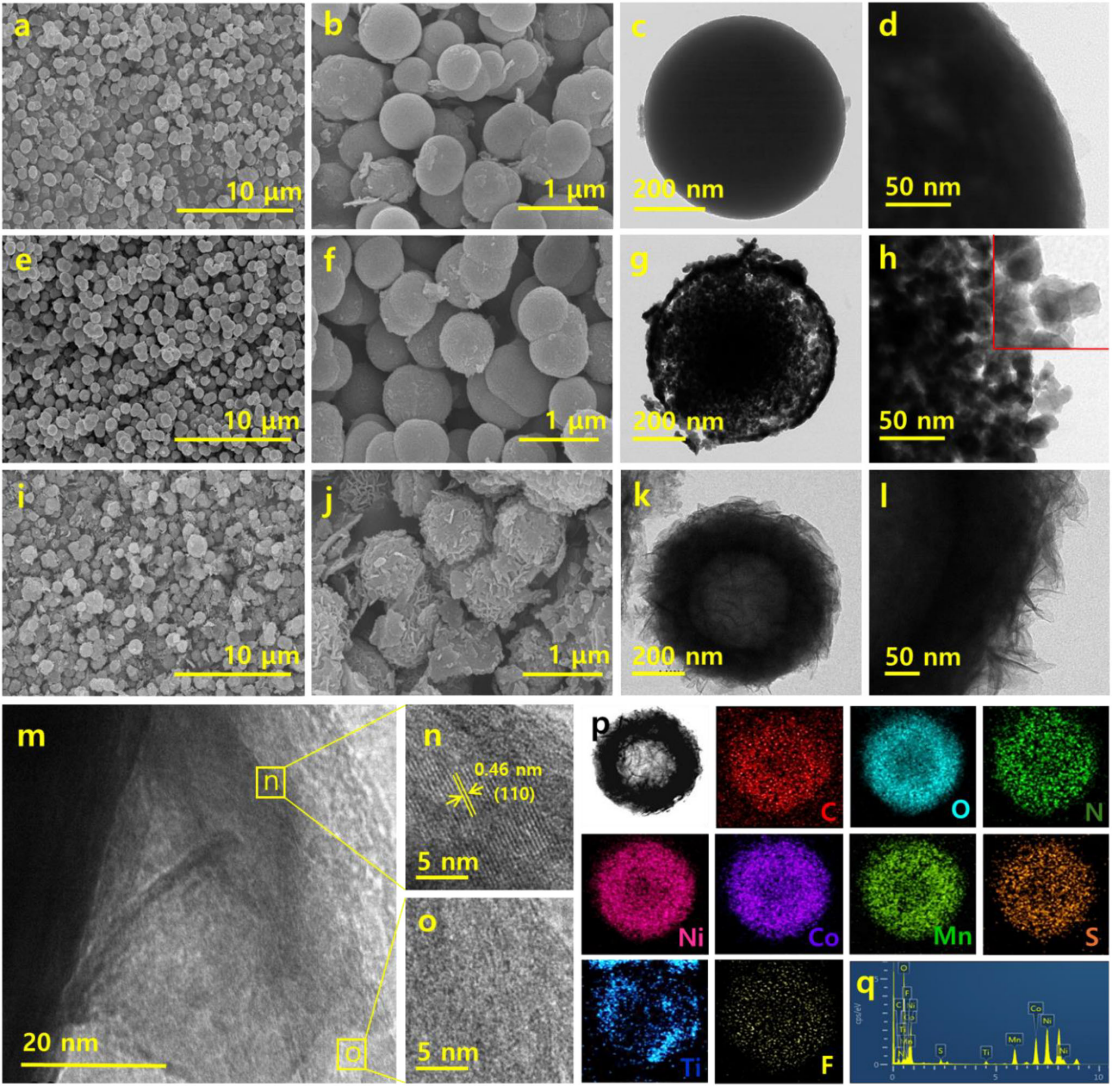
a,b) SEM images and c,d) TEM and HRTEM images of NiCoMn‐glycerate. e,f) SEM images and g,h) TEM and HRTEM images of NiCoMn‐S spheres. i,j) SEM images and k,l) TEM images of MXe/NiCoMn‐LDH/S hollow spheres. m–o) HRTEM images of the MXe/NiCoMn‐LDH/S hollow spheres. p) HAADF‐STEM images, EDS mapping images, and q) EDS spectra of MXe/NiCoMn‐LDH/S.

The SEM images in Figure 1a,b show the uniform, monodisperse nanospheres of NiCoMn‐glycerate with a diameter of ≈800 nm. The TEM images (Figure 1c,d) further demonstrate that the nanospheres have a solid core with a multi‐grain assembly. Moreover, the high‐resolution image in Figure 1d displays an amorphous structural nature on the surface of the solid sphere. The energy dispersive spectroscopy (EDS) spectra (Figure S1, Supporting Information) further show that the Ni, Mn, Co, and O elements were distributed on the mixed metal composite sample. The atomic ratio of the Ni, Co, and Mn was 3.0:0.8:0.9, and the color of the samples was light brown. By inducing the sulfidation process for 8 h on the NiCoMn glycerate system, the morphology of the spheres was preserved, with rough and porous surface features (Figure 1e,f). As the time of the sulfidation process increased, the color of the sample changed to black, indicating the formation of metal sulfides.

The TEM image (Figure 1g) of the 8 h sulfidated samples shows a porous structural assembly with a slightly increased diameter and a well‐defined hollow interior structure. The hollow structural formation is due to the Kirkendall effect during the sulfidation process. The HRTEM image (insert of Figure 1h) of the NiCoMn‐S shows that the surface is amorphous, and the thickness of the shell wall is ≈50 nm. The EDS spectra and elemental mapping analysis by HAADF‐STEM confirmed the existence of Ni, Co, Mn, and S elements, with an atomic ratio of ≈1:1:0.1 (Figure S2, Supporting Information). Compared to NiCoMn‐glycerate, the Mn content was reduced in the NiCoMn‐S sample, which can be attributed to the higher solubility of Mn species during the sulfidation process. Due to the highly favorable dissolution of Mn during the sulfidation process, the shell wall of mixed metal sulfides is not uniform and exhibits some porous features. Figure S3 (Supporting Information) further demonstrates that, concerning sulfidation reaction time, the expansion of the hollow structure in the system occurs. The static analysis results showed that the average thickness of the shell is ≈50 nm. The SEM images (Figure S4, Supporting Information) of the layered structure confirm the successful synthesis of Ti_3_C_2_T_x_ MXene via selective etching of the Al layer from Ti_3_AlC_2_ MAX phase using an acid etching process. As depicted in Figure S5 (Supporting Information), the delaminated Ti_3_C_2_T_x_ resulted in a small, thin, layered sheet‐like structure after the sonication process. In the both stages, F ions were presence on the layered surface which is due to the residual LiF based sources. However, F ion ratio was reduced after delamination process as compared the stacked MXene surface. The TEM results confirmed that the few‐layer MXene nanosheets with basal planes formed a crystalline network of MXene nanosheets. Adding the well‐dispersed MXene nanosheets during the sulfidation process resulted in the successful assembly of Ti_3_C_2_T_x_ sheets on the surface of NiCoMn‐S hollow spheres through the electrostatic self‐assembly (Figure 1i,j). The specific interaction of MXene on the surface of NiCoMn‐glycerate favors the specific shell growth and leads to a 3D hierarchical hollow sphere assembly with the surface integrity of nanosheet arrays (Figure 1l). The lattice spacing of Ti_3_C_2_T_x_ is ≈0.46 nm, corresponding to the (110) planes, with the amorphous surface showcasing the MXene tagging on the NiCoMn‐S surface (Figure 1m,n). Furthermore, some regions of the layered surfaces exhibited an amorphous phase (Figure 1o), which likely contributed to the formation of mixed metal LDH based derivatives. Subsequent structural investigations validated that the hierarchical layered array on NiCoMn‐S was indeed the composite interface of NiCoMn‐LDH and MXene. Due to the formation of NiCoMn‐LDH and MXene with NiCoMn‐S, the shell wall thickness resulted in a diameter of ≈120 nm, featuring a hollow core and a hierarchical shell with a thicker shell wall. The self‐assembled MXe/NiCoMn‐LDH/S hollow spheres were characterized using EDS spectra (Figure S6, Supporting Information) and mapping (Figure 1p), integrating the dimensions of MXene and NiCoMn‐LDH/S, which may be described as a 2D‐3D interface. The distribution of Ti and F on the outer shell surface, while Ni, Co, Mn, O, and S were uniformly distributed throughout the shell wall, confirmed the hollow structure of MXene‐wrapped NiCoMn‐LDH/S spheres. The zeta potential values of NiCoMn‐glycerate, NiCoMn‐S, MXene, and MXe/NiCoMn‐LDH/S were measured to elevate the electrostatic interactions, as summarized in Table S1 (Supporting Information). The positively charged NiCoMn glycerate has a high tendency to interact with the MXene nanosheets on its surface due to the electrostatic interaction. Later during the sulfidation process, the leached‐out metal ions, which have a positive charge, were more likely to interact with the MXene surface through electrostatic attraction and chemical bonding at the functional active sites on the MXene surface. In the meantime, the ion exchange process was favorable to form the mixed metal sulfide on the shell wall of NiCoMn‐glycerate resulting in the successful formation of MXe/NiCoMn‐LDH/S hollow spheres. In the absence of MXene, there is no formation of LDH nanosheet arrays, which results in the favorable interaction of dissolved mixed metal nanostructures during the ion exchange process over the surface‐tagged MXene nanosheets and feasibly forms the NiCoMn‐LDH on the shell wall of the nanospheres.

**Figure 2:**
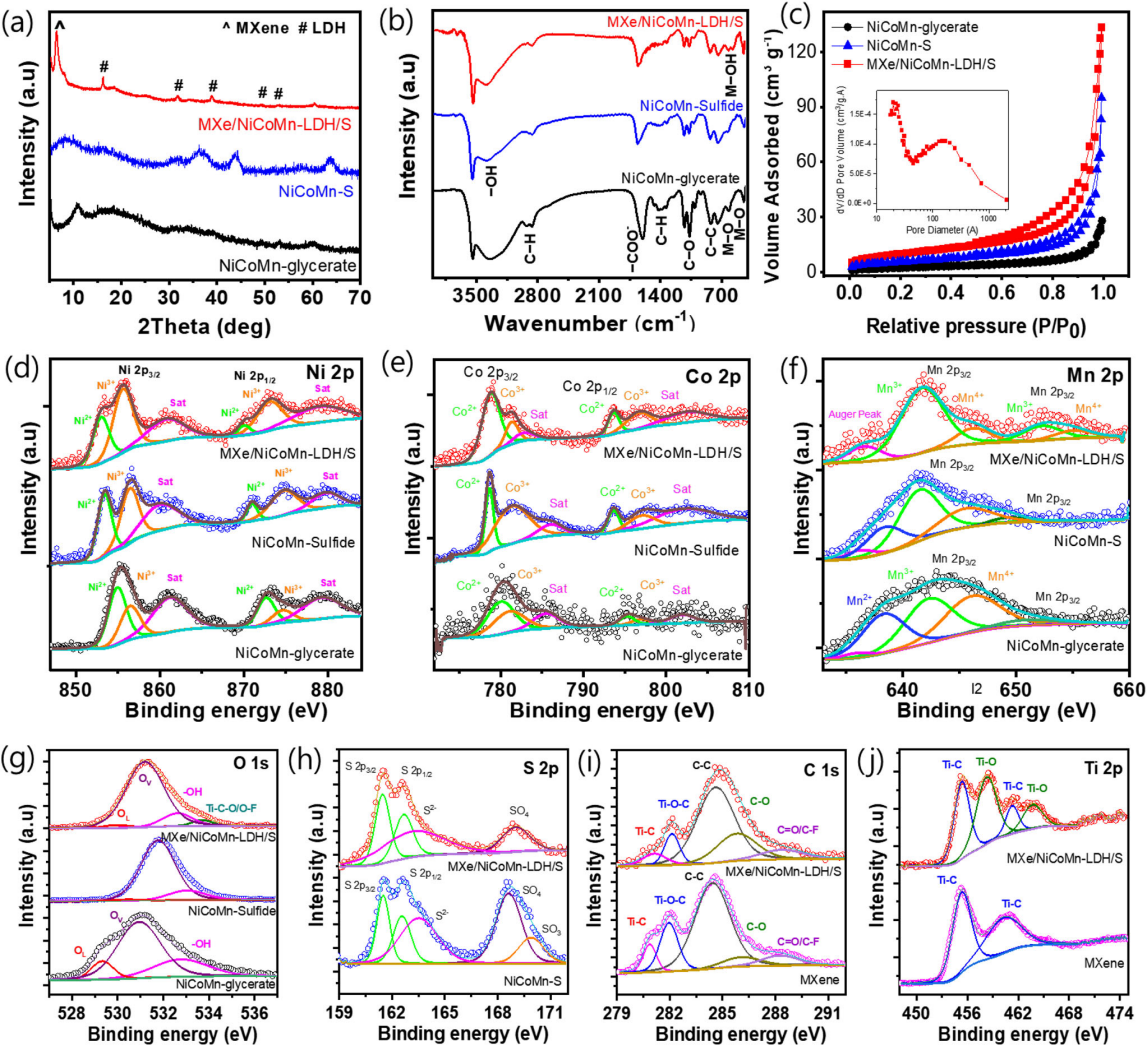
a) XRD patterns, b) FTIR spectra, and c) N_2_ adsorption‐desorption isotherm of the pristine NiCoMn‐glycerate, NiCoMn‐S, and MXe/NiCoMn‐LDH/S composites. The high‐resolution XPS spectra of d) Ni 2p, e) Co 2p, f) Mn 2p, g) S 2p, and h) O 1s for the NiCoMn‐glycerate, NiCoMn‐S, and MXe/NiCoMn‐LDH/S composites. i) C 1s and j) Ti 2p for the MXene and MXe/NiCoMn‐LDH/S composites.

To explore the transformation of mixed metal glycerate, the XRD of the NiCoMn‐glycerate, NiCoMn‐S, and MXe/NiCoMn‐LDH/S were compared in **Figure** **2**a. For the mixed metal glycerate, the broad peaks appeared at 10.7°, which is characteristic of alkoxides and is correlated with the previous reports.^[^ ^37^ ^]^ After sulfidation, the XRD pattern exhibits diminished glycerate‐related peaks and no other distinct crystal diffraction patterns, indicating the formation of an amorphous phase of mixed‐metal sulfide hollow spheres. MXene prepared from the acid etching of MAX resulted in the diffraction peaks at 8.8°, 18.1°, indicating the successful etching of MXene from MAX (Figure S7, Supporting Information). Furthermore, after the delamination of MXene, the (002) peak shifted from 8.8° to 7.8°, indicating an increase in interlayer spacing. The existence of MXene‐related peaks in the mixed‐phase sulfide sample confirms the successful formation of MXene‐NiCoMn sulfide composites. Additionally, the diffraction peaks at 16.1°, 18.3°, 24.8°, 31.7°, 34.9°, 31.7°, 39.1°, 49.3°, 52.9°, and 60.6° correspond to the mixed phase of LDH‐related structure, which can be attributed to Mn(OH)_2_ (JCPDS No. 073–1133), hexagonal Co(OH)_2_ (JCPDS No. 001–0357), and hexagonal Ni(OH)_2_ (JCPDS No. 14–0117) indicating the formation of mixed‐metal hydroxide‐based nanocomposites.^[^ ^38^, ^39^ ^]^ The integration of MXene as an in situ tag at 80 °C resulted in the favoring of LDH‐related peaks on the composite surface. In solvothermal prepared samples, LDH‐related peaks are absent, apart from the mixed sulfide and MXene‐based diffraction peaks with the disordered morphology (Figure S8, Supporting Information), indicating that the low‐temperature reflux reaction with in situ tagging of MXene favors LDH growth.

The presence of glycerates on the samples was further confirmed through the FTIR analysis (Figure 2b). In the as‐prepared NiCoMn‐glycerate, a broad band emerged at ≈3360 and ≈3600 cm^−1^ which is related to the stretching vibration O−H hydroxyl groups. The adsorption bands at ≈2869 and ≈2936 cm^−1^ originated from the stretching vibrations of C−H, respectively. The peak ∼≈1600 cm^−1^ were corresponds to the −COO^−^ asymmetric stretching vibrations and the broad band between the 1300–1500 cm^−1^ were assigned to the C−H bending vibrations. The peaks at ≈1100 and ≈800 cm^−1^ originated from the C−O stretching and C−C stretching vibrations, respectively, indicating the presence of glycerate, which is consistent with previous reports.^[^ ^40^ ^]^ The peak at ≈689 and ≈588 cm^−1^ are related to the metal‐oxygen stretching vibration peaks (Ni−O, Co−O, and Mn−O).^[^ ^41^ ^]^ After the sulfidation process, the glycerate‐related peak intensity decreased, indicating the successful transformation of mixed metal glycerate into metal sulfide. The composite sample exhibited new peaks ≈593 and 551 cm^−1^, which correspond to the bending vibrations of M−OH bonds in the composite samples. Furthermore, the Raman spectra of the composite samples with sulfidation time showed that the A_1g_ and E_g_ vibrations associated with Ti and C atoms occurred at the initial stage due to the tagging of MXene. Later, the metal‐oxygen‐metal vibration states became stronger, resulting in mixed metal‐LDH growth on the composite surface (Figure S9, Supporting Information). The presence of MXene and LDH vibrational states on the MXe/NiCoMn‐LDH/S surface confirms a composite interface with rich oxidation capabilities. The specific surface area of the NiCoMn‐glycerate, NiCoMn‐S, and MXe/NiCoMn‐LDH/S was obtained by the N_2_ adsorption‐desorption isotherm (Figure 2c). NiCoMn‐glycerate and NiCoMn‐S hold the surface area of 12.453 and 20.674 m^2^ g^−1^, respectively. In comparison, the MXe/NiCoMn‐LDH/S process has a small surface area of 29.321 m^2^ g^−1^. The enlarged surface area and pore volume of MXe/NiCoMn‐LDH/S, at ≈0.09674 cm^3^ g^−1^, are due to the hierarchical hollow structural assembly, which provides more accessible active reaction sites for the Faradaic reaction, thereby promoting reaction kinetics. ICP‐OES was used to characterize the composition of MXe/NiCoMn‐LDH/S (Table S2, Supporting Information), showing that the MXene tagging minimizes the dissolution of Mn on the spherical assembly that would promote the reaction active sites of the hierarchical hollow composite.

The XPS was analyzed to examine the chemical composition and valence states of the heterostructured hollow spheres. The survey spectrum (Figure S10, Supporting Information) confirms the presence of Ni, Co, Mn, O, S, and Ti, indicating successful integration of the constituent elements in MXe/NiCoMn‐LDH/S composite. The high‐resolution Ni 2p spectrum (Figure 2d) of the MXe/NiCoMn‐LDH/S composite, peaks at 853.1 and 870.1 eV are assigned to Ni^2+^ 2p_3/2_ and Ni^2+^ 2p_1/2_, while the peaks at 855.6 and 873.2 eV manifest the presence of Ni^3+^ 2p_3/2_ and Ni^3+^ 2p_1/2_, respectively.^[^ ^40^ ^]^ Additionally, two satellite peaks at 860.9 and 879.2 eV are observed. The shift in peak positions confirms the formation of an LDH/sulfide interface on the catalytic surface. Surface composition analysis of Ni (Table S3, Supporting Information) further reveals a high concentration of Ni on the MXe/NiCoMn‐LDH/S composite spheres. Similarly, the Co 2p XPS spectrum (Figure 2e) displays two sets of doublets with satellite peaks, corresponding to Co^2+^ and Co^3+^ oxidation states. The peaks at 778.8 and 793.7 eV are assigned to Co^2+^ 2p_3/2_ and Co^2+^ 2p_1/2_, while the peaks at 780.8 and 797.2 eV correspond to Co^3+^ 2p_3/2_ and Co^3+^ 2p_1/2_, along with the two satellite peaks at 787.1 and 802.2 eV, respectively.^[^ ^30^, ^31^ ^]^ Upon examining the integrity of MXene, a shift in peak position toward higher binding energy was observed, indicating strong interactions between MXene, LDH, and the mixed‐metal sulfide surface, as evidenced by the NiCoMn‐S sample. The Mn 2p spectra of the MXe/NiCoMn‐LDH/S sample (Figure 2f) were deconvoluted into two main peaks corresponding to Mn 2p_3/2_ and Mn 2p_1/2_, which were further resolved into four sub‐peaks. The peaks at 641.8 and 652.5 eV are attributed to Mn^3+^, while the peaks at 646.4 and 655.5 eV confirm the presence of Mn^4+^.^[^ ^25^ ^]^ Compared to the mixed‐metal sulfides, the MXe/NiCoMn‐LDH/S sample exhibits stronger Mn‐related energy states, suggesting enhanced stability of Mn‐related functionality on the hybrid hollow spheres. The observed peak shifts further indicate the transformation of Mn–S/Mn–OH during the ion exchange process. Moreover, the introduction of MXene caused noticeable shifts in the Ni 2p, Co 2p, and Mn 2p peaks of the MXe/NiCoMn‐LDH/S composite, evidencing strong electronic interactions and enhanced electron transfer across the composite surface. The O 1s spectra (Figure 2g) revealed contributions from lattice oxygen (O_L_), oxygen vacancies (Ov), and chemisorbed –OH groups. Compared to the NiCoMn glycerate sample, the MXene‐containing composite showed a diminished O_L_ signal alongside Ov states, indicating the presence of defect‐related functionalities within the mixed‐metal sulfides. Following the introduction of MXene, the composites exhibited lattice oxygen (O_L_)‐related energy states due to the formation of mixed‐metal LDH structures. Additionally, new peaks located at 533.2 and 533.8 eV correspond to Ti–C–Ox and O–F functionalities, respectively, resulting from the interaction between MXene and the surrounding environment. The high‐resolution S 2p spectrum (Figure 2h) of the MXe/NiCoMn‐LDH/S was deconvoluted into three main components. The peaks observed at 161.5 and 162.6 eV are attributed to S 2p_1/2_ and S 2p_3/2_, respectively, signifying the existence of metal–sulfur bonds. A peak at 163.7 eV represents the S^2−^ species, while an additional peak at 168.6 eV addresses the surface sulfur with high oxidation such as SO_4_^−^ in the composites.^[^ ^31^ ^]^ The MXe/NiCoMn‐LDH/S resulted in stronger metal–sulfur bonds than the NiCoMnS, indicating the improved structural integrity of the composite assembly. The C 1s spectrum of MXe/NiCoMn‐LDH/S (Figure 2i) shows five fitted peaks corresponding to Ti−C (281.1 eV), O−Ti−C (282.1 eV), C−C (284.6 eV), C−O (286.4 eV), and C═O/C−F (289.1 eV) bonds. A reduction in the Ti−C peak intensity suggests partial oxidation of MXene during the formation of mixed metal sulfide hollow spheres. The Ti 2p spectra (Figure 2j) of pristine MXene and MXe/NiCoMn‐LDH/S composite further support this observation. The doublet peaks at 455.3 and 461.6 eV correspond to the Ti−C bonds, confirming the crystalline nature of MXene.^[^ ^42^ ^]^ A new peak at 458.6 eV in the composite is attributed to Ti−O bonding, indicating partial oxidation of MXene due to its strong interaction with the mixed‐metal sulfides. The MXe/NiCoMn‐LDH/S was generated via mixed interfaces of metal–sulfur and SO^4−^‐based surface functionality with the various mixed metal oxidation states. However, Ni and Co based oxidization states are look somehow similar in the MXe/NiCoMn‐LDH/S and NiCoMnS samples, but the presence of dominant Mn^3+^ states on the composite interface due to the LDH growth could facilitate the electronic properties and improve the sensitivity of the composite assembly.

### 2.2 Electrode Fabrication and Nitrite Sensing on Modified Electrodes

**Figure 3:**
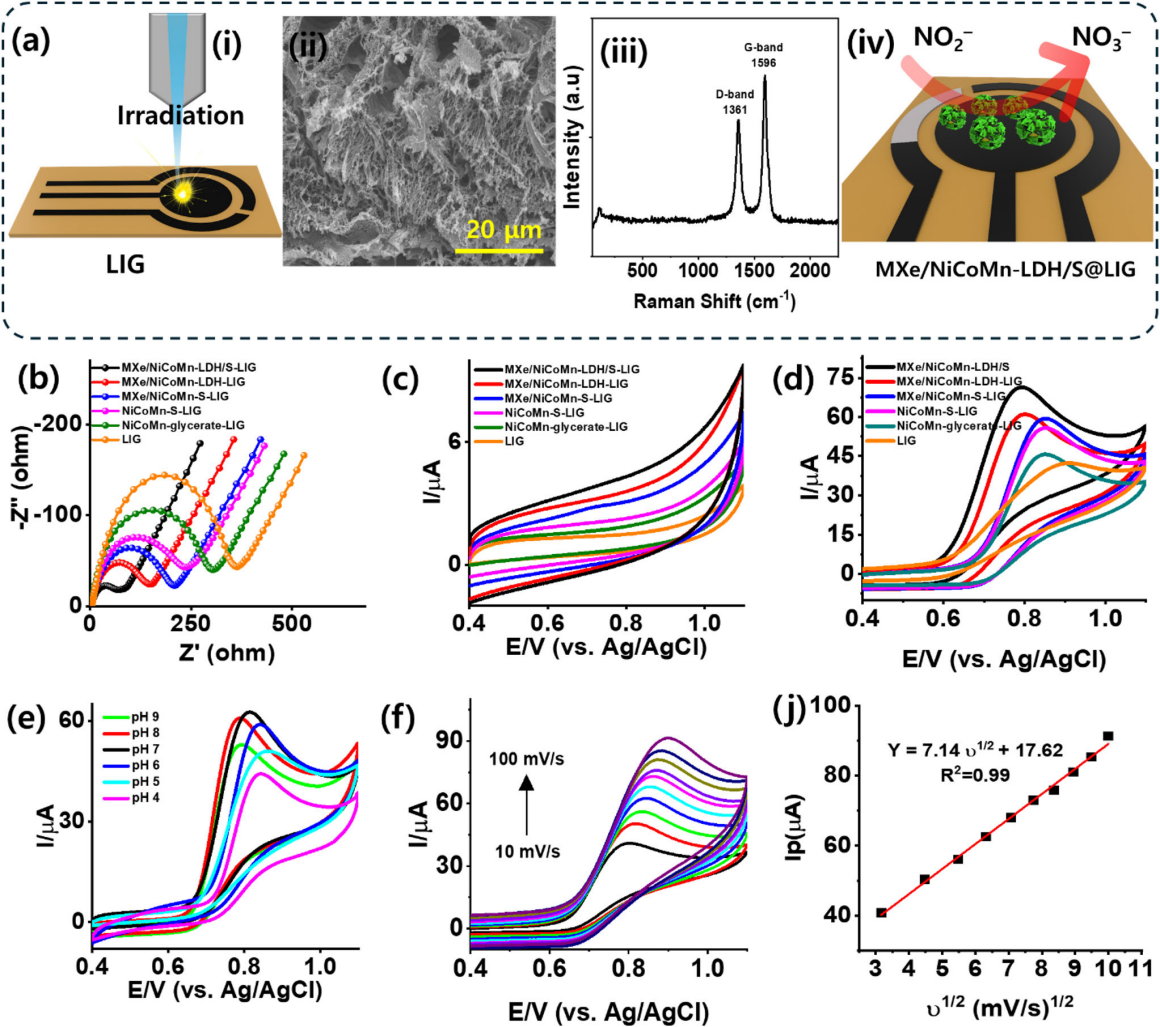
a) Fabrication strategy and structural characteristic of LIG electrode: i) Schematic illustration of LIG fabrication, ii) SEM, iii) Raman spectra, and iv) schematic illustration of modified MXe/NiCoMn‐LDH/S‐LIG electrode. b) Comparative EIS spectra of the modified electrodes in 0.1 M KCl containing 1 mM of Fe(CN)_6_^3−/4−^. The CVs of the modified electrodes in PBS (pH = 7) c) before and d) after the addition of NO_2_^−^, e) CV responses behavior of MXe/NiCoMn‐LDH/S‐LIG electrode to NO_2_^−^ oxidation in different pH values. f) CV curves of the MXe/NiCoMn‐LDH/S‐LIG electrode in different scan rates in the presence of 500 µM of the NO_2_^−^. j) Corresponding plot of peak current versus square root of scan rate.

To study the electrochemical performance of the MXe/NiCoMn‐LDH/S composite, a three‐electrode system was designed using a LIG electrode on a flexible polyamide substrate. The schematic illustration of the LIG preparation process is shown in **Figure** **3**a(i). LIG is a mask‐free strategy for preparing 3D electrodes through high‐quality graphene‐like structures, which offer enhanced electrical conductivity and excellent electrochemical stability.^[^ ^43^ ^]^ SEM image of the optimized, highly porous LIG substrate is shown in Figure 3a(ii). The interconnected nanowire like texture with porous topography of LIG significantly increase the surface area, thereby facilitating faster electron transfer and enhancing the interaction between the composites. This structure contributes to improved sensing performance, offering high stability and reproducibility. The average Raman spectra (Figure 3a(iii)) of the optimized LIG exhibits the characteristics of the D, G, and 2D bands. Initially, the structural quality and sheet resistance of the LIG electrodes were characterized through Raman and current–voltage (*I–V*) measurements, and the preferred LIG electrode is optimized for further studies (Figure S11, Supporting Information). The optimized LIG electrode exhibits a high density of induced defect state and a multilayered graphene interface, as confirmed by the notable I_D_/I_G_ ratio of 0.81 ± 0.05 and the I_2D_/I_G_ ratio of 0.51 ± 0.01. FTIR analysis (Figure S12, Supporting Information) further confirmed the existence of functional groups on the LIG surface, including C–H stretching (2997 cm^−1^), C═O carbonyl groups (1709 cm^−1^), and C–O bonds (1234 cm^−1^), indicating surface oxygen functionalities. The wettability of the prepared LIG was studied through contact angle measurements, which yielded a contact angle of θ = 32° ± 2° (Figure S13, Supporting Information), indicating a more hydrophilic surface than that of the commercial GCD electrodes (θ = 77° ± 3°). This enhanced hydrophilicity is likely attributed to the oxygen‐based functional groups on the LIG surface. Figure 3a(iv) illustrates the schematic illustration of the integrated hybrid MXe/NiCoMn‐LDH/S hollow sphere on the 3D LIG electrode surface.

First, the interfacial electrochemical behavior and electron transfer kinetics of the different modified electrodes, such as MXe/NiCoMn‐LDH/S‐LIG, MXe/NiCoMn‐LDH‐LIG, MXe/NiCoMn‐S‐LIG, NiCoMn‐S‐LIG, NiCoMn‐glycerate‐LIG, and bare LIG, were investigated by the electrochemical impedance spectroscopy (EIS) technique in 0.1 M KCl containing 1 mM of Fe(CN)_6_^3−/4−^. The Nyquist plot (Figure 3b) consists of the semicircular portion in the higher frequency region, which refers to the electron transfer process. The equivalent electrical circuit of EIS data was fitted by the Randles circuit (Figure S14, Supporting Information), where *Z*_w_, *R*_ct_, *C*_dl_, and *R*_s_ represent the Warburg impedance, charge transfer resistance, double layer capacitance, and solution resistance, respectively. The diameter of the semicircle for the final modified electrode was smaller compared to other modified electrodes, suggesting a lower charge transfer resistance. The fitting results address the *R*_ct_ value of the modified electrodes as 74.5, 153.9, 211.5, 241.7, 305.2, and 361.0 Ω for the MXe/NiCoMn‐LDH/S‐LIG, MXe/NiCoMn‐LDH‐LIG, MXe/NiCoMn‐S‐LIG, NiCoMn‐S‐LIG, NiCoMn‐glycerate‐LIG, and bare LIG, respectively. It can be suggested that the synergistic effects of the MXe/NiCoMn‐LDH/S composition facilitated a faster electron tunneling between the electrode surface and Fe(CN)_6_^3−/4−^. The enlarged surface area of the MXe/NiCoMn–LDH/S heterostructure effectively facilitates sensitive and rapid electron transfer, enabling the high conductivity on the composite surface.

The standard electron transfer rate (K^°^) of the different electrodes was obtained by *R*_ct_ value according to Equation 1.

(1) $$K^{0}=\frac{\mathrm{RT}}{n^{2}F^{2}R_{\mathrm{Ct}}\mathrm{AC}}\;$$

In which the R, T, n, F, *R*_ct_, A, and C are gas constant (8.314 J K^−1^ mol^−1^), temperature (298 K), number of the transfer electron, Faraday's constant (96 486 C mol^−1^), charge transfer resistance (Ω), electrode surface area (cm^2^), and Fe(CN)_6_^3−/4−^ concentration (1 mM), respectively. The obtained K^°^ (cm/s) values were 4.78 × 10^−7^, 2.31 × 10^−7^, 1.68 × 10^−7^, 1.47 × 10^−7^, 1.17 × 10^−7^, and 0.986 × 10^−7^ for MXe/NiCoMn‐LDH/S‐LIG, MXe/NiCoMn‐LDH‐LIG, MXe/NiCoMn‐S‐LIG, NiCoMn‐S‐LIG, NiCoMn‐glycerate‐LIG, and bare LIG, respectively. These data confirmed that MXe/NiCoMn‐LDH/S has high conductivity and superior electrocatalytic features for transferring electrons, making it a promising material for nitrate oxidation.

The electrocatalytic activity toward NO_2_^–^ was analyzed by comparing the cyclic voltammetry (CV) curves of the modified electrodes taken in PBS solution (pH 7.0) in the absence (Figure 3c) and presence (Figure 3d) of NO_2_^–^. As shown in Figure 3c, the background current of the electrodes was gradually increased by modification of LIG with NiCoMn‐glycerate, NiCoMn‐S, MXe/NiCoMn‐LDH, MXe/NiCoMn‐S, and MXe/NiCoMn‐LDH/S, respectively, without having any redox peaks. This signifies the enhancement of the electroactive surface area of the LIG with different electroactive composites. The sensitivity of the electrodes toward NO_2_^−^ (500 µM) is shown in Figure 3d. The bare LIG exhibited a weaker oxidation signal (≈0.90 V), which can be attributed to sluggish electron‐transfer kinetics and significant surface fouling. Moreover, the modified electrodes with MXe/NiCoMn‐LDH and MXe/NiCoMn‐S enhance the oxidation signal and shift the oxidation potential to a more negative value, thereby improving the overall oxidation responses. The NiCoMn‐glycerate and NiCoMn‐S did not exhibit any significant changes in the oxidation responses. Surprisingly, the hybridized MXe/NiCoMn‐LDH/S electrode exhibited an oxidation potential of ≈0.81 V, along with a significantly higher oxidation current compared to other electrode sets. This enhancement can be attributed to the integration of MXene with mixed LDH and sulfide phases, which effectively reduces the oxidation potential and promotes the current density. Furthermore, compared to the traditional glassy carbon electrode, the LIG‐tagged composite electrode has yielded a high current density, which signifies the high specific surface area of LIG, promoting high sensitivity to electroactive composites for NO_2_^–^ detection (Figure S15, Supporting Information). The electrochemical data indicate that the hollow MXe/NiCoMn‐LDH/S electrode exhibits an increased electroactive surface area and swift electron transfer, positioning it as a strong contender for NO_2_^−^ detection. Moreover, it offers several analytical advantages, including outstanding electrocatalytic performance and efficient charge transport. These attributes highlight its potential for effective implementation in a wide range of practical applications. To further optimize the sensing performance of MXe/NiCoMn‐LDH/S, the effect of the electrolyte pH, as a primary parameter for detecting NO_2_^−^, was investigated by CV over different pH values from 4 to 9. As shown in Figure 3e, the oxidation peak current of NO_2_^−^ in 0.1 M PBS gradually increased as the pH was raised from 4 to 7. Additionally, the oxidation potential shifted to a more negative value of ≈0.82 V, indicating enhanced electrooxidation of NO_2_^–^ and more efficient electron transfer under alkaline conditions. Further, it suggested that in more acidic media, the oxidation peak current of NO_2_^–^ is lower than at pH 7 due to the conversion of NO_2_^–^ to the NO and NO_3_^−^, as well as increased protonation of NO_2_^−^. However, the electrooxidation of NO_2_^–^ decreased as the pH was further elevated to 9. At higher pH levels, nitrite oxidation was hindered due to an insufficient proton supply.^[^ ^44^ ^]^ Further, in the neutral pH, the LDH component maintains structural integrity, minimizing metal dissolution and the functional groups of MXene were effectively protonated at this pH, enhancing electrolyte wettability. The synergistic effects on the composite interface at the neutral pH improve the adsorption, electron transport, and redox processes, resulting in the superior electrochemical performance of the composite at pH 7. Therefore, pH 7 was selected as the optimal electrolyte condition for the electrooxidation of NO_2_^−^ on the surface MXe/NiCoMn‐LDH/S. Additionally, the impact of different scan speeds was examined to elucidate the NO_2_^−^ sensing mechanism and reaction kinetics at the surface of the hybridized MXe/NiCoMn‐LDH/S electrode (Figure 3f). Figure 3j shows that the NO_2_^−^ anodic peak current rose linearly with the square root of the scan rate from 10 to 100 mV/s, suggesting that the electrochemical process was regulated by diffusion‐based mechanism. The linear regression equation was I_Pa_ (µA) = 7.14 × υ^1/2^ + 17.62, with a correlation coefficient of R^2^ = 0.99. The electrocatalytic oxidation process can be expressed as follows:

(2) $${{\mathrm{NO}}_{2}}^{-}+H_{2}O\;\to {{\mathrm{NO}}_{3}}^{-}+2H^{+}+2e^{-}$$

The relationship between the anodic peak current (I_Pa_) and the square root of the scan rate (υ^1/2^) is depicted in Figure 3j to evaluate the kinetic mechanism according to the Randles–Ševčík equation. The linear increase in the anodic current with the square root of the scan rate confirms that the electrochemical charge‐transfer process is dominated by diffusion rather than adsorption, indicating a stable and reversible redox behavior of NO^−2^ on the modified electrode surface.

### 2.3 NO_2_^−^ Detection by Linear Sweep Voltammetry (LSV), Chronoamperometry (CA)

**Figure 4:**
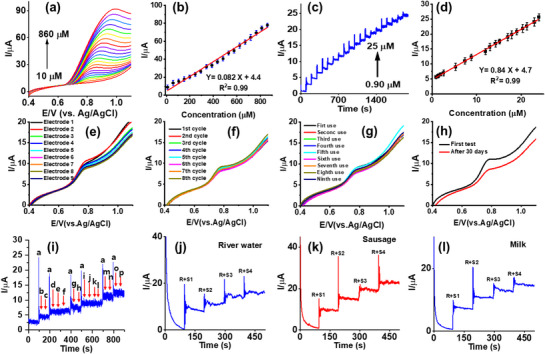
a) LSV calibration of NO_2_^−^ oxidation from 10 to 860 µM, b) calibration curve of LSV, c) Chronoamperometric calibration of NO_2_^−^ oxidation from 0.90 to 25 µM, d) calibration curve of Chronoamperometric, e) reproducibility, f) repeatability, g) reusability, h) long term stability after 30 days stability analysis i) interference study in the presence of 5 µM NO_2_^−^, 1000‐fold excess of b–h) Fe^2+^, Cu^2+^, Mn^3+^, Ca^2+^, Zn^2+^, Na^+^, and Br^−^ and i–p) 50‐fold excessive concentrations of glucose, sucrose, uric acid, hydroquinone, catechol, ascorbic acid, H_2_O_2_, and paracetamol. Determining the NO_2_^−^ in different real samples of j) river water, k) sausage, and l) milk.

Moreover, LSV as another sensitive technique used for the measurement of NO_2_^−^ concentration quantitatively on the surface MXe/NiCoMn‐LDH/S in PBS solution at pH 7.0. **Figure** **4**a is the LSV response of the MXe/NiCoMn‐LIG/S‐LIG, which shows that the anodic current response has increased along with the NO_2_^−^ concentration in the range from 10 to 860 µM. Figure 4b illustrates that the peak current value is directly proportional to the concentration of NO_2_^−^, and this connection is linear with increasing concentration. The fitting resulted in the linear regression equation as I_p_ (µA) = 0.082 × + 4.4 (R^2^ = 0.99). Based on the WHO's recommended threshold range of 8.7–28.3 µM for nitrate in drinking water, the MXe/NiCoMn‐LDH/S sensor's low detection limit becomes pertinent for the effective detection of NO_2_^−^ oxidation. Furthermore, CA is a sensitive technique for checking the electrooxidation of NO_2_^−^ on the surface of MXe/NiCoMn‐LDH/S in a PBS solution at pH 7.0 at low concentrations. The CA technique was employed to investigate the electrochemical signal of NO_2_^−^ on the modified rotating disk electrode with MXe/NiCoMn‐LDH/S across various injected NO_2_^–^ concentrations at a fixed (0.8 V) potential (Figure 4c). As depicted in Figure 4d, the calibration curve by CA shows the linearity from 0.90 to 25 µM. Based on the NO_2_^–^ oxidation current, the linearity was gained with the equation of I_p_ (µA) = 0.84 × + 4.7 (R^2^ = 0.99). The MXe/NiCoMn‐LDH/S sensor showed a broad linear range, and the lowest LOD limit by CA was calculated as 0.21 µM using the standard formula for LOD = 3.3σ/S, where S is the slope and σ is the standard deviation of the amperometry signal for the blank (three runs). Meanwhile, the sensitivity was found to be 11.88 µA µM^−1^ cm^−2^. Based on these results, it can be concluded that the MXe/NiCoMn‐LDH/S‐LIG system exhibits excellent sensitivity for nitrite detection. Compared to previously reported nitrite‐sensitive electrodes (as shown in **Table** **1**), the MXe/NiCoMn‐LDH/S electrode demonstrated exceptional detection performance, characterized by an extensive linear detection range, remarkable sensitivity, and an exceedingly low LOD. The sensor's capability to accurately measure nitrite levels confirms that the MXe/NiCoMn‐LDH/S electrocatalyst is effective, implying a wide range of practical applications to ensure environmental and human safety.

**Table 1:** Comparison of electrochemical sensing parameters on different platforms for the detection of nitrite.

Electrode	method	Linear range [µM]	LOD [µM]	Sensitivity [µA µM^−1^ cm^−2^]	Refs.
OMCF	CA	1–6000	0.1	998.57	[12]
Ms‐Au/ZnO@Pt‐CC	CA	0.2–4986	0.09	5677	[13]
Pd‐Cu‐Mo_2_C‐GCE	CA	0.005–0.165	0.00035	3.308	[44]
AuNPs/GCE	DPV	10‐3800	2.4	–	[45]
Au@CQDs‐MXene/GCE	DPV	1‐500 500‐3200	0.078	–	[46]
LIG/f‐MWCNT‐AuNPs	SWV	10–140	0.9	–	[47]
ns‐ZnO/N‐rGO/GCE	CA	0.037–5900	0.08	301.9	[48]
Cu_x_OGCS‐BPPG	CV	10‐100	2.038	1500	[49]
SnO_2_/Pt/Ti/SiO_2_/Si	CA	10‐400	1.7	22.56	[50]
GNPs‐SC‐CPE	CA	1.0–150	0.4	–	[51]
BioAc/Cc	CA	0.35–478.21	0.015	1946.54	[52]
MoS_2_/C_3_N_4_/GCE	DPV	0.1–1100	0.065	0.0439	[53]
Pd@Bi_2_S_3_MS/SPE	DPV	0.01–500	0.0033	3660	[54]
NiCo_2_O_4_/GCE	CA	10–300	1.04	1.03	[55]
Co_3_O_4_/CC	CV	1–4000	0.12	3053	[56]
MXe/NiCoMn‐LDH/S‐LIG	CA	0.90 – 25	0.21	11.88	This work
	LSV	10 – 860	7.38	1.16	

### 2.4 Reliability and Selectivity of the Electrode

The sensing ability of the electrodes depends on several analytical parameters, including reproducibility, repeatability, and reusability. These parameters were checked using LSV with 70 µM NO_2_^−^ in 0.1 M PBS. Nine separate electrodes were fabricated and tested for the electrochemical sensing of 50 µM NO_2_^−^ in 0.1 M PBS. The relative standard deviation (RSD) was determined to be 1.49%, indicating high reproducibility (Figure 4e). Eight successive measurements of one MXe/NiCoMn‐LDH/S‐LIG electrode for the NO_2_^–^ oxidation yielded RSD of 2.45%, representing excellent repeatability (Figure 4f). Moreover, the electrode was fabricated, showing RSD of 3.39%, indicating high reusability (Figure 4g). Finally, the long‐term stability of the MXe/NiCoMn‐LDH/S was measured after 30 days via LSV in 0.1 M PBS. After this period, the anodic peak current remained at 80.2% of its initial value, indicating exceptional stability (Figure 4h). The electrode was stored at a temperature of 4 °C for a duration exceeding 30 days.

To evaluate the selectivity of the MXe/NiCoMn‐LDH/S electrode for NO_2_^–^ detection in the presence of common organic and inorganic interferences, CA was employed. As shown in Figure 4i, the sensing performances of the MXe/NiCoMn‐LDH/S electrode were evaluated in the presence of possible ionic interferences like Fe^2+^, Cu^2+^, Mn^3+^, Ca^2+^, Zn^2+^, Na^+^, and Br^–,^ each at 1000‐fold excessive concentrations than NO_2_^−^ (5 µM). Moreover, the 50‐fold excessive concentrations of organic compounds, such as glucose, sucrose, uric acid, hydroquinone, catechol, ascorbic acid, H_2_O_2_, and paracetamol, did not affect the anodic signal of NO_2_^–^ detection, with a deviation below 5%. These results confirm that the MXe/NiCoMn‐LDH/S electrode exhibits high selectivity for NO_2_^–^ detection, even in the presence of numerous interferences. Therefore, the MXe/NiCoMn‐LDH/S electrode can be a promising electrode for detecting NO_2_^−^ in complex solutions of real samples.

### 2.5 NO_2_^−^ Quantitative Analysis in Real Samples

As shown in Figure 4j–l, the electrochemical response of the MXene/NiCoMn‐LDH/S electrode increased remarkably with the successive addition of NO_2_^−^ spike solutions from 5 to 20 µM in real river water, sausage, and milk samples through the standard addition method. This pronounced enhancement in the oxidation current clearly demonstrates the high sensitivity and excellent electrocatalytic performance of the proposed electrode, even in complex real sample matrices. The recovery experiments were conducted using river water, sausage extract, and milk samples to further evaluate the reliability and applicability of the sensor in practical conditions. The average recoveries obtained were 94.3%–106.6% for river water, 97.0%–102.0% for sausage, and 99.0%–107.8% for milk samples, all RSDs below 5%. These results indicate the high accuracy and reproducibility of the developed sensor for NO_2_^−^ detection in real samples (**Table** **2**). Moreover, the corresponding plots of current response versus NO_2_^−^ concentration for each sample type exhibited excellent linearity (Figure S16, Supporting Information), confirming the robust analytical performance and strong ability of the MXene/NiCoMn‐LDH/S electrode for NO_2_^−^ detection in diverse environmental and food matrices.

**Table 2:** The NO_2_‐ recovery determination from different real samples.

Real sample	Spike [µM]	Found [µM]	Average recovery [%]	RSD [%]
River water	0	0	–	–
	5	5.33	106.6	2.1
	10	9.43	94.3	4.2
	15	15.14	100.9	3.6
	20	20.1	100.5	3.7
Sausage	0	0	–	–
	5	4.85	97.0	2.4
	10	10.20	102.0	3.9
	15	15.00	99.99	4.2
	20	19.92	99.56	4.4
Milk	0	0	–	–
	5	4.52	99.04	4.6
	10	10.78	107.8	3.4
	15	14.86	99.80	3.6
	20	19.83	99.90	4.2

**Figure 5:**
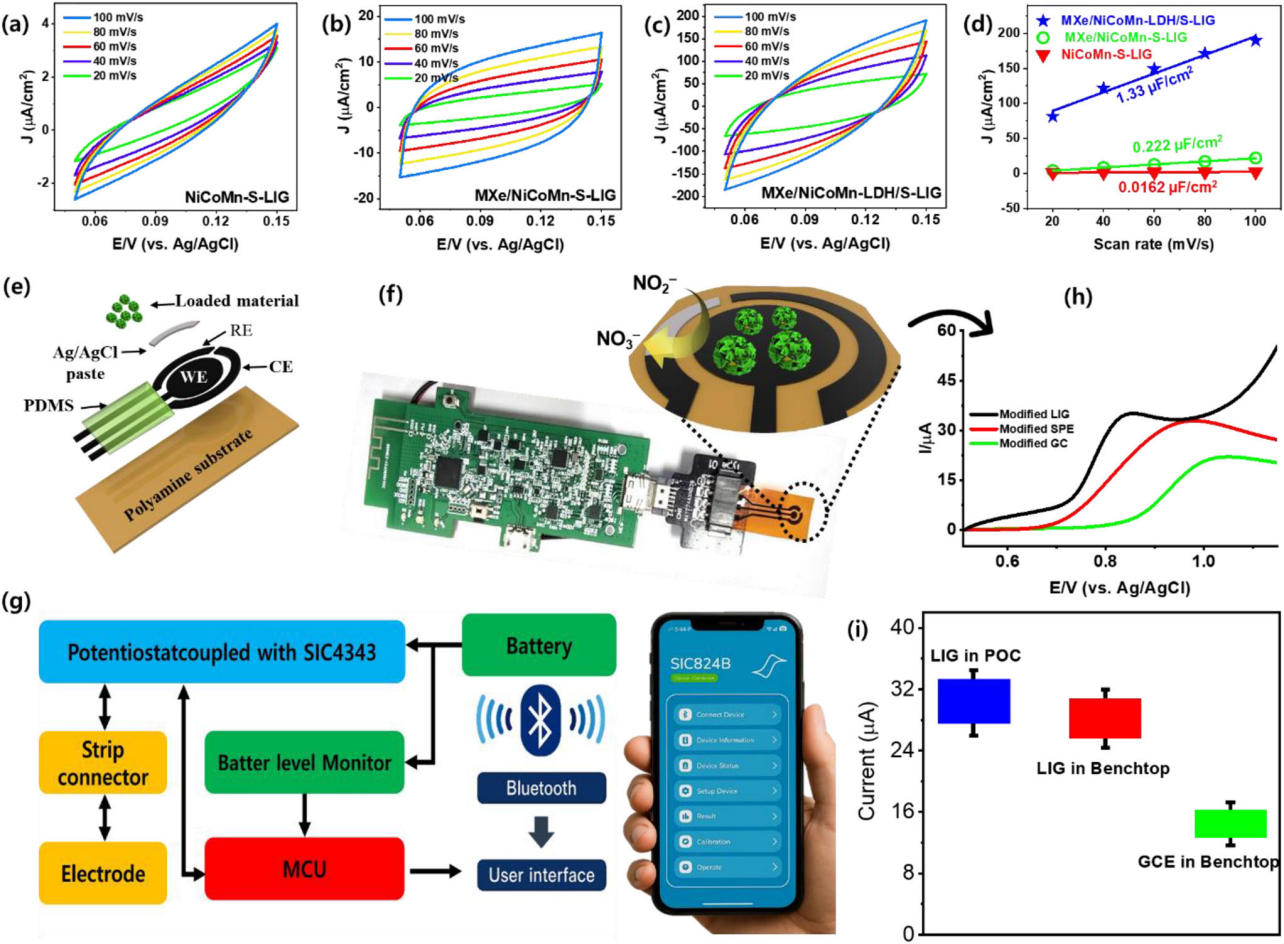
Estimating the electrochemical surface area of the a) NiCoMn‐S, b) MXe/NiCoMn‐S, and c) MXe/NiCoMn‐LDH/S electrodes by performing the CV response at different scan rates in 1 M KOH electrolyte. d) The evaluated *C*_dl_ is obtained by plotting the current density versus sweep rate. e) Schematic diagram of the components of a flexible MXe/NiCoMn‐LDH/S‐LIG‐based sensor platform for on‐site detection. f) Overview image of smartphone‐based electrochemical system, and g) the key block diagram of the detector. h) Detecting nitrite from the oxidation of CV peak currents on the LIG‐based electrode on a smartphone. f) Compared with the peak current signal between real water pollutants for the benchtop and portable potentiostats.

To address the active sites and the electrochemical surface area (ECSA) of the different modified electrodes, the CV was performed in 1 M KOH solution at different scan rates within a non‐Faradaic potential range of 0.05 to 0.15 V. **Figure** **5**a–c illustrate the CV curves of NiCoMn‐S‐LIG, MXe/NiCoMn‐S‐LIG, and MXe/NiCoMn‐LDH/S‐LIG electrodes at various scan rates. The peak current at the potential of 0.10 V of the modified electrodes was fitted linearly with the scan rate and presented in Figure 5d. The double‐layer capacitance (*C*_dl_) value of the modified electrodes was represented from the slope value of the linear plot, where the *C*_dl_ values of NiCoMn‐S‐LIG, MXe/NiCoMn‐S‐LIG, and MXe/NiCoMn‐LDH/S‐LIG were 0.0162, 0.222, and 1.332 µF cm^−2^, respectively. The ECSA, which is proportional to the *C*_dl_, indicates that the hybridized MXe/NiCoMn‐LDH/S‐LIG exhibits a higher ECSA of 0.751 cm^−2^ than others, correlating with the specific surface area calculated from the N_2_ adsorption‐desorption isotherm. The results showed that the specific surface area and the ECSA of the MXe/NiCoMn‐LDH/S‐LIG are higher than those of NiCoMn‐S, which provides high surface interactive sites with high electrochemical performances. With its high electrochemical surface area and heteroatom electronic configuration, the system typically undergoes electron transfer, modulating the local electronic states of the transition metal centers and influencing the dissipation and aggregation of charges at the interface. The differential charge density of the NiCoMn‐LDH/S with MXene integrity facilitates the effective transfer of electrons on the surface, thereby enhancing the catalytic ability. Validating the potential advantage of MXe/NiCoMn‐LDH/S toward the high electrochemical sensitivity of NO_2_^−^, the POC system with the composed Android smartphone support was designed with an electrochemical detection module (EDM) controlled by a microcontroller unit (MCU). The schematic diagram of the flexible MXe/NiCoMn‐LDH/S loaded LIG electrode is shown in Figure 5e. The overall design and the black diagram of the POC system design are shown in Figure 5f,g. The POC system consisted of a flexible electrode and a handheld detector, coupled with an Android application loaded on a smartphone. The handheld detector module (SIC824B) is composed of a unit assembly that includes a MCU, electrode socket, battery management module, SIC4343 potentiostat unit, and Bluetooth module. The handheld detector can be taken to remote areas, and with the help of wireless connectivity via a Bluetooth interface with a smartphone, electrochemical performance can be analyzed, and the results are displayed in real‐time through the Android application. The hybridized LIG electrode was utilized as a sensor for NO_2_^−^ detection in both a traditional benchtop model and a POC system. In the POC system, the LSV signals were constantly recorded, and the detection of NO_2_^−^ monitoring is shown in Figure 5h. For a comparative analysis, the 15 real water samples were collected as background samples to monitor the NO_2_^−^ detection, which was directly measured through the portable and benchtop instruments with LIG and GCE electrodes. However, it is essential to note that the current signal from the LIG‐based electrodes is higher than that of the traditional GCE‐based electrodes, resulting in the high sensitivity of the LIG surface, which offers portable and flexible advantages. However, the GCE and LIG modified electrodes have the same thermodynamic potential; the redox potential has shifted slightly due to the overpotential of different electrodes. The peak current of the modified LIG electrode in the POC, as well as in the benchtop, is higher than that of traditional GCE‐based electrodes. The NiCoMn‐LDH/S‐LIG‐based hybridized electrode exhibits a two‐fold increase in peak current, confirming its superior electrical conductivity and electrochemically active surface area, which is discussed throughout the study. Being a low‐cost, flexible sensing platform with high current response, the LIG‐based hybrid electrodes have addressed the need for high sensitivity and selectivity in the electrochemical detection of NO_2_^−^, which will aid in various real‐time environmental and food industry applications.

## 3 Conclusion

In this study, the solvothermal method, followed by a sulfidation‐based anion exchange reaction with subsequent functionalization of MXene, was used to fabricate hybridized MXe/NiCoMn‐LDH/S hollow spheres. Incorporating the smartphone's inbuilt, portable, hybridized electrode on the LIG substrate as a flexible electrochemical sensor highlights the high sensitivity and selectivity toward the detection of NO_2_– in an aqueous environment. The experimental results demonstrate the synergistic effect of the mixed metal LDH/sulfide at the MXene surface interface, which enhances the adsorption capacity and provides a high density of electrochemically active sites, thereby promoting the electrochemical performance of the modified electrode. Notably, developed nitrite sensors exhibit rapid electron transfer kinetics and a swift response to nitrite, delivering excellent analytical performance with a LOD of 0.21 µM using CA, and a wide linear range from 0.90 to 25 µM. It exhibits a high sensitivity of 11.88 µA µM^−1^ cm^−2^, corresponding to the large ECSA provided by the MXe/NiCoMn‐LDH/S heterostructure. Furthermore, under LSV measurements, the sensor achieved an extended linear range of 10 to 860 µM with a LOD of 7.38 µM and a sensitivity of 1.15 µA µM cm^−^^2^, along with excellent stability and strong anti‐interference capability, highlighting its versatility for nitrite detection across a broad concentration range. Compared to traditional bench‐top electrochemical workstations, the self‐developed, cost‐effective, portable LIG‐based electrodes with a wireless smartphone‐controlled potentiostat offer a significant advantage, characterized by a strong NO_2_^−^ signal with satisfactory recovery rates in real samples. The exploration of new hybrid materials integrated with in‐built artificial intelligence is expected to enhance wireless electrochemical sensing performance, providing greater accuracy and a promising pathway for analyzing other analytes in practical food samples and healthcare contexts.

## Conflict of Interest

The authors declare no conflict of interest.

## Supporting information



Supporting Information

## Data Availability

The data that support the findings of this study are available on request from the corresponding author. The data are not publicly available due to privacy or ethical restrictions.
