# Supplementary material for: Smartphone‐Assisted Wireless Ultrasensitive Nitrite Detection in Food Samples via Hierarchical MXene/NiCoMn‐LDH/Sulfide Heterostructure on Flexible Laser‐Induced Graphene Electrode
Source: Small. 2025 Nov 12;21(51):e10411. doi: 10.1002/smll.202510411 (PMC12723330; doi:10.1002/smll.202510411)
Supplement: Supplementary file 1 — Supporting Information [file SMLL-21-e10411-s001.docx]

**Supporting Information**

**Smartphone-assisted wireless ultrasensitive nitrite detection in food samples via hierarchical MXene/NiCoMn-LDH/sulfide heterostructure on flexible laser-induced graphene electrode**

*Kugalur Shanmugam Ranjith^a*^, Ali Mohammadi^a^, A. T. Ezhil Vilian^a^,* *Yonghyeon Park^b^, Ganji Seeta Rama Raju^a^, Yun Suk Huh^b,*^, Young-Kyu Han^a,*^*

^a^Department of Energy and Material Engineering, Dongguk University-Seoul, Seoul 04620, Republic of Korea

^b^Department of Biological Sciences and Bioengineering, Nano Bio High-Tech Materials Research Center, Inha University, Incheon 22212, Republic of Korea

^*^Corresponding authors: [ranjuphy@gmail.com](mailto:ranjuphy@gmail.com) (K. S. Ranjith), yunsuk.huh@inha.ac.kr (Y. S. Huh), ykenergy@dongguk.edu (Y.-K. Han)

# **1. Experimental materials and methods**

## **1.1 Materials**

Nickel(II) nitrate hexahydrate (Ni(NO_3_)_2_·6H_2_O ≥ 99.9%), cobalt nitrate hexahydrate (Co(NO_3_)_2_·6H_2_O ≥ 98%), manganese(II) nitrate tetrahydrate (MnNO_3_)_2_·4H_2_O ≥ 97%), isopropanol (IPO), glycerol, Nafion® (5%,w/w), thioacetamide (TAA ≥ 98%), lithium fluoride (LiF), hydrochloric acid (HCl), isopropanol (IPO), tetramethylammonium hydroxide solution (TMAOH, 25%), and sodium nitrite (NaNO_2_ ≥ 99.0%) were obtained from Sigma-Aldrich, USA. Titanium aluminum carbide (Ti_3_AlC_2_, MAX, 300 mesh) powder was bought from Carbon-Ukraine Ltd (Ukraine). All reagents were of analytical grade and used without further purification. The electrolyte used in the experiments was phosphate-buffered saline (PBS). PBS solutions with varying pH values were prepared by combining 0.2 M Na_2_HPO_4_ and 0.2 M NaH_2_PO_4_ stock solutions in different proportions.

## **1.2 Preparation of polymetallic modified electrodes**

### **1.2.1 Preparation of laser-induced graphene (LIG)**

The LIG electrode pattern was designed using CorelDraw software, and the design was fabricated with a computer-controlled laser engraving system (VLS2.30, Universal Laser Systems, USA) to irradiate a polyamine (PI) substrate with a thickness of 0.2 mm. First, the PI substrate was cleaned and wiped with ethanol, and then it was fixed on a heat-resistant substrate to ensure it was clean and flat. Before fabricating the desired pattern, the LIG power and scan speed were optimized to attain a conductive, defect-enriched, stable substrate. Finally, the desired pattern was engraved on the PI substrate using a laser power of 1.5 W and a speed of 63.5 mm/s. The laser has a wavelength of 10.6 μm with a beam spot size of 76 μm, and the distance between the laser and the substrate is 8 mm. The prepared LIG substrate was cut to an appropriate size, insulated with the Kapton film to control the LIG reactive area, and preserved in a vacuum box for further use.

### **1.2.2 Fabrication of layered MXene**

Typically, 1 g of LiF was added to 20 mL of 9 M HCl and stirred for 10 min. Ti_3_AlC_2_ MAX (1 g) was then gradually added and stirred for 72 h at 40 °C [**1**]. The resulting material was repeatedly suspended in distilled (DI) water and centrifuged until the pH reached 6.0. The final suspension was ultrasonicated and centrifuged at 5000 rpm for 5 min; the supernatant was then collected, freeze-dried, and subsequently subjected to the delamination process. Briefly, 100 mg of freeze-dried MXene was dispersed in 40 mL of TMAOH under constant stirring for 12h, then redispersed in 40 mL of DI water. The mixture was sonicated for 4h in an ice bath under an Ar atmosphere and subsequently centrifuged at 3000 rpm for 10 min. The supernatant nanosheets were then collected, freeze-dried, and used to create the hybrid heterostructure.

### **1.2.3** **Fabrication of Ni-Co-Mn glycerate spheres**

To prepare the Ni-Co-Mn glycerate nanospheres, 365.7 mg (25 mmol) of Ni(NO_3_)_2_·6H_2_O, 392.8 mg (25 mmol) of Co(NO_3_)_2_·6H_2_O, and 338.9 mg (25 mmol) of Mn(NO_3_)_2_·4H_2_O, were dissolved in 54 ml of IPO and stirred for 5 min. Glycerol (16 mL) was added to the above mixture and stirred for 60 min to achieve a homogeneous dispersion. The reactant was then transferred to a Teflon-lined stainless-steel autoclave and maintained at 200°C for 10 h. After the reaction was cooled to room temperature, the product was collected, centrifuged, and washed several times with ethanol. It was then dried at 80°C for 6 h and named NiCoMn-glycerate.

### **1.2.4 Fabrication of MXene-tagged NiCoMn-LDH/S hollow spheres**

To fabricate hollow NiCoMn-LDH/S spheres, 100 mg of the as-prepared NiCoMn-glycerate was first dispersed in 30 mL of ethanol. Then, 100 mg of thioacetamide (TAA) was added to the solution, followed by stirring for 5 minutes. The resulting mixture was transferred to a reflux system and maintained at 80 °C for 8 hours. The resulting product was collected by centrifugation, washed several times with ethanol and deionized water, and then freeze-dried overnight. The final product is referred to as NiCoMn-LDH/S spheres. To prepare the MXene-based composite electrode, the above-mentioned similar processes were used. During the synthesis of the NiCoMn-sulfide spheres, once the reaction reached 80 °C, single-layered MXene was gradually introduced into the system. Specifically, 10 mg of MXene (dispersed in ethanol at a concentration of 1 mg/mL) was slowly added under mild stirring. After maintaining the reaction at 80 °C for 8 hours, the system was cooled to room temperature. The slow addition of MXene into the reaction environment effectively induced electrostatic interactions on the surface of the NiCoMn-glycerate spheres, leading to the formation of surface-interactive sites that enhance the stability of the shell wall. This facilitated the formation of hierarchical hollow mixed metal sulfide spheres while preserving the structural integrity of the MXene. During the ion exchange process at elevated temperatures, the partially dissolved mixed metal glycerate interacted with the MXene-functionalized surface, promoting the in-situ formation of layered double hydroxides (LDH) on the shell surface. Although the solvothermal method is generally considered optimal for preparing hollow NiCoMn-sulfide structures [**2**], the above reflux-based method was adopted due to the difficulty of slowly introducing MXene under solvothermal conditions. Structural and morphological analyses revealed the presence of a layered double hydroxide (LDH) interface combined with metal sulfide nanograins, facilitated by the incorporation of MXene. Accordingly, the resulting composite is denoted as MXene/NiCoMn-LDH/S. The slow addition of MXene in the reaction environment has effectively created electrostatic interactions on the NiCoMn-glycerate sphere surface, forming surface interactive sites that promote the stability of the shell wall. This, in turn, enables the formation of hierarchical hollow mixed metal sulfide spheres with MXene integrity. The dissolved mixed metal glycerate during the ion exchange process was reacted with the MXene-tagged surface at an elevated temperature, which was favorable for the formation of LDH on the shell surface. For comparative analysis, MXe/NiCoMn-S, NiCoMn-S, NiCoMn-glycerate, and MXe/NiCoMn-LDH composites were prepared through the solvothermal reaction, and the electrochemical properties were investigated. The detailed experimental procedure was included in the supporting information. The fabrication strategy of the MXe/NiCoMn-LDH/S and electrode modification on LIG substrate for further use is illustrated in **Scheme 1**.

## **1.3 Material Characterization**

Morphologic and structural analyses of the prepared MXe/NiCoMn-LDH/S electrodes were performed by high-resolution scanning electron microscopy (HRSEM, SU 8010; Hitachi), high-resolution transmission electron microscopy (FETEM, JEM-2100F; JEOL), and selected-area electron diffraction (SAED). X-ray diffraction (XRD) patterns were obtained using a PANalytical X'Pert Pro multipurpose X-ray diffractometer using Cu Kα irradiation (λ = 0.15406 nm). Fourier transform infrared (FTIR) spectrometry (JASCO FTIR-6600) was used to detect surface functionalities in the range of 400 to 4000 cm^−1^. Raman spectroscopy was performed using a 532-nm laser Raman microscope (FEX, NOST) and X-ray photoelectron spectroscopy (XPS, Thermo Scientific spectrometer) with an Al Kα (1486.6 eV) source. The binding energies were corrected using the carbon 1s peak at 284.6 eV, and XPS peaks were fitted using Casa XPS Software. Brunauer-Emmett-Teller (BET) surface areas and N_2_ adsorption at 77°K were measured using a Tristar ASAP 2020 unit to determine specific surface areas and pore size distributions. Trace amounts of CAP were measured using an HPLC system (Shimadzu Perkin Elmer Optima 7300 DV). The pH of the solution was tested using a pH meter (SevenCompact S213) with an accuracy of ±0.01.

## **1.4 Electrochemical detection of NO_2_^–^**

All electrochemical methods in this study utilized a three-electrode working system. The fabricated three-electrode design of the LIG electrode consisted of a working electrode (area: 0.0706 cm^2^), a counter electrode (area: 0.2863 cm^2^), and a reference electrode (area: 0.1802 cm^2^), all of which were connected by an electrical path. The LIG had a sheet resistance of around 21 Ω/sq. The Ag/AgCl ink was coated on the reference electrode surface. The end connecting pads are covered with silver epoxy to prevent the electrode damage from the physical force of connecting clips. The fabricated MXe/NiCoMn-LDH/S was dispersed in ethanol (1 mL), and 20 μL of Nafion (5% w/w) was added to the dispersion, which was then drop-cast onto the working electrode area on the LIG substrate. The modified electrode was dried under vacuum for 30 min to prepare the MXe/NiCoMn-LDH/S-LIG. The MXe/NiCoMn-LDH/S-LIG, MXe/NiCoMn-LDH-LIG, MXe/NiCoMn-S-LIG, NiCoMn-S-LIG, and NiCoMn-glycerate-LIG electrodes were fabricated in the same way. Electrochemical measurements, including cyclic voltammetry (CV), linear sweep voltammetry (LSV), chronoamperometry (CA), and electrochemical impedance spectroscopy (EIS), were conducted using a workstation (model number: CHI 7089E). The LSV was performed with a potential range of 0.4 V to 1.1 V and a scan rate of 50 mV/s. The i-t method is primarily used to analyze sensor response times, where a NO_2_^–^ solution was added to the electrolyte at 50 s intervals after the current stabilized under an applied voltage of 0.8 V. The electrolyte and NO_2_^–^ solutions were prepared in 0.1 M PBS, and a 500 mM NO_2_^–^ solution was used for electrochemical detection and optimization of experimental parameters.

## **1.5 Pretreatment of real samples**

The real samples were prepared according to the previously reported procedure [**3**]. Briefly, river water samples were collected from the Han River, South Korea, using 1 L glass bottles and stored at 4 °C until analysis. Before the electrochemical measurements, the samples were filtered through a 0.5 μm PTFE membrane (Mitex, Millipore) to remove suspended particulates. Sausage samples (5.0 g) were homogenized with 12.5 mL of saturated borax solution and boiled for 30 min. After slight cooling, 2.5 mL of a 30% ZnSO_4_ solution was added, and the mixture was heated at 80°C for 10 minutes to precipitate the proteins. The resulting mixture was cooled to room temperature, filtered, and diluted to 50 mL with 0.1 M PBS (pH 7.0). Milk samples were purchased locally and diluted tenfold with 0.1 M PBS (pH 7.0) to minimize matrix interference. For all real samples, known concentrations of nitrite were spiked using the standard addition method to assess recovery and quantify NO_2_⁻ levels. The pH of each prepared solution was carefully measured and adjusted to the optimal value of 7.0 using a calibrated pH meter, ensuring consistent ionic strength and enhanced current response.

**1.6 Designing and fabricating a POC testing system**

The POC testing system was coupled with an electrochemical detection module supplied by Silicon Graft Technology PLC, controlled by a microprocessor (MCU) and supported by an Android smartphone, as shown in the **Scheme. 1d**. The 3.7 V lithium battery powers the modules, and an SIC824B module regulates the power, coupled with a built-in Bluetooth module, making the POC a wireless system to couple with a smartphone.

**1.7. Predicting the diffusion-controlled mechanism**

In electrochemistry, electron transfer between the solution and the electrode involves two main processes: the faradaic process, which is associated with charge transfer involving the analyte, and the non-faradaic process, which is related to the charging and discharging of the electrical double layer at the electrode–electrolyte interface. To evaluate the behavior of the faradaic process, the Randles–Ševčík equation is commonly employed. This equation helps determine whether the electrochemical reaction is diffusion-controlled by analyzing the relationship between the peak current ($I_{p}$) and the square root of the scan rate ($v^{1/2}$). When a linear correlation is observed between $I_{p}$and $v^{1/2}$, it confirms that the electrochemical process is governed by diffusion. At 25 °C, the Randles–Ševčík equation is expressed as:

$$I_{p}=2.69\times{10}^{5}\text{ }n^{3/2}AD^{1/2}Cv^{1/2}$$

where $I_{p}$is the peak current (A), $n$is the number of electrons transferred, $A$is the electrode surface area (cm²), $D$is the diffusion coefficient (cm^2^·s⁻^1^), $C$is the analyte concentration (mol·cm⁻^3^), and $v$is the scan rate (V·s⁻^1^). In our study, the relationship between the anodic peak current ($I_{pa}$) and the square root of the scan rate ($v^{1/2}$) is expressed as $I_{pa}(\mu A)=7.14\text{ }v^{1/2}+17.62$, with a correlation coefficient ($R^{2}$) of 0.99. The excellent linearity of this plot indicates that the electrochemical process is predominantly diffusion-controlled, confirming that electron transfer between the analyte and the electrode surface follows the diffusion mechanism described by the Randles–Ševčík equation.

The non-faradaic region of the cyclic voltammograms was utilized to determine the electrochemically active surface area (ECSA) of the fabricated electrodes. Cyclic voltammetry (CV) measurements were recorded at various scan rates ranging from 20 to 100 mV·s⁻^1^. From these non-faradaic CVs, the capacitive current density was calculated using $\Delta J=(J_{a}-J_{c})/2$at a fixed potential, where $J_{a}$and $J_{c}$represent the anodic and cathodic current densities, respectively. A plot of $\Delta J$versus scan rate ($\nu$) yields a straight line, and the slope corresponds to the double-layer capacitance ($C_{dl}$) according to $i_{c}=C_{dl}\nu$. Finally, the ECSA was calculated using $\text{ECSA}=C_{dl}/C_{s}$, where $C_{s}$is the specific capacitance of a smooth surface of the same material. According to these formulas, we calculate the diffusion control process of nitrite oxidation as the ECSA of all composite materials.

**Reference**

1. [Kugalur Shanmugam Ranjith](https://pubs.rsc.org/en/results?searchtext=Author%3AKugalur%20Shanmugam%20Ranjith),  [Sonam Sonwal](https://pubs.rsc.org/en/results?searchtext=Author%3ASonam%20Sonwal), [Ali Mohammadi](https://pubs.rsc.org/en/results?searchtext=Author%3AAli%20Mohammadi), [Ganji Seeta Rama Raju](https://pubs.rsc.org/en/results?searchtext=Author%3AGanji%20Seeta%20Rama%20Raju), [Mi-Hwa Oh](https://pubs.rsc.org/en/results?searchtext=Author%3AMi-Hwa%20Oh), [Yun Suk Huh](https://pubs.rsc.org/en/results?searchtext=Author%3AYun%20Suk%20Huh), and  [Young-Kyu Han](https://pubs.rsc.org/en/results?searchtext=Author%3AYoung-Kyu%20Han), Imparting hydrophobicity to a MOF on layered MXene for the selective, rapid, and ppb level humidity-independent detection of NH_3_ at room temperature, ***J. Mater. Chem. A***, 12 (**2024**), 26132-26146, <https://doi.org/10.1039/D4TA04656K>.
2. Yan Zhou, Yongsheng Fu, Tingting Zhang, Chenyao Hu, Fen Qiao, Junfeng Wang, Hyuk-Jun Noh, Jong-Beom Baek, Synthesis of size-controllable, yolk-shell metal sulfide spheres for hybrid supercapacitors, [***Chemical Engineering Journal***](https://www.sciencedirect.com/journal/chemical-engineering-journal), [476](https://www.sciencedirect.com/journal/chemical-engineering-journal/vol/476/suppl/C) (**2023**), 146377, <https://doi.org/10.1016/j.cej.2023.146377>.
3. Ramesh Madhaiyan, Devabharathi Vijayaraghavan, Srinithi Shankar, Umamatheswari Seeman, Nagoor Meeran Mohamed Ibrahim, Sankar Chinnusamy, Fabrication of spinel NiCo_2_O_4_ nanoflowers by simple hydrothermal method for effective electrochemical detection of NO_2_^−^ in processed food sample, [***Food Chemistry***](https://www.sciencedirect.com/journal/food-chemistry), 480 (**2025**), 143964, <https://doi.org/10.1016/j.foodchem.2025.143964>.


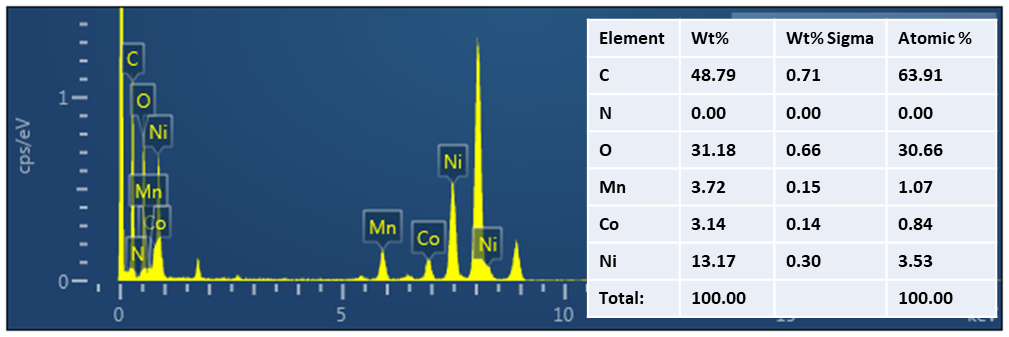


**Fig. S1** EDAX spectra of the as-prepared NiCoMn-glycerate.

**
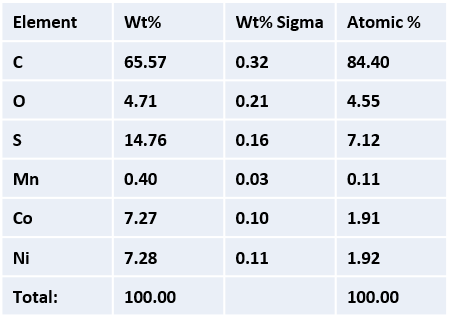

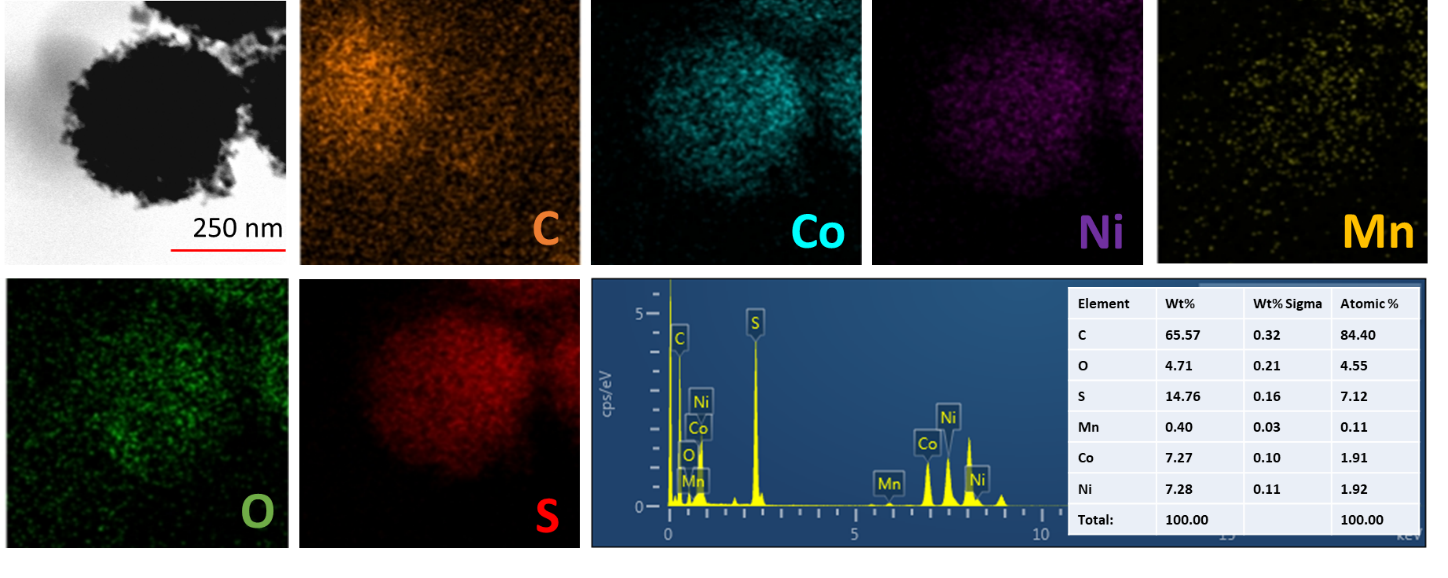
**

250 nm

**Fig. S2** EDAX spectra and mapping of NiCoMn-S nanospheres.


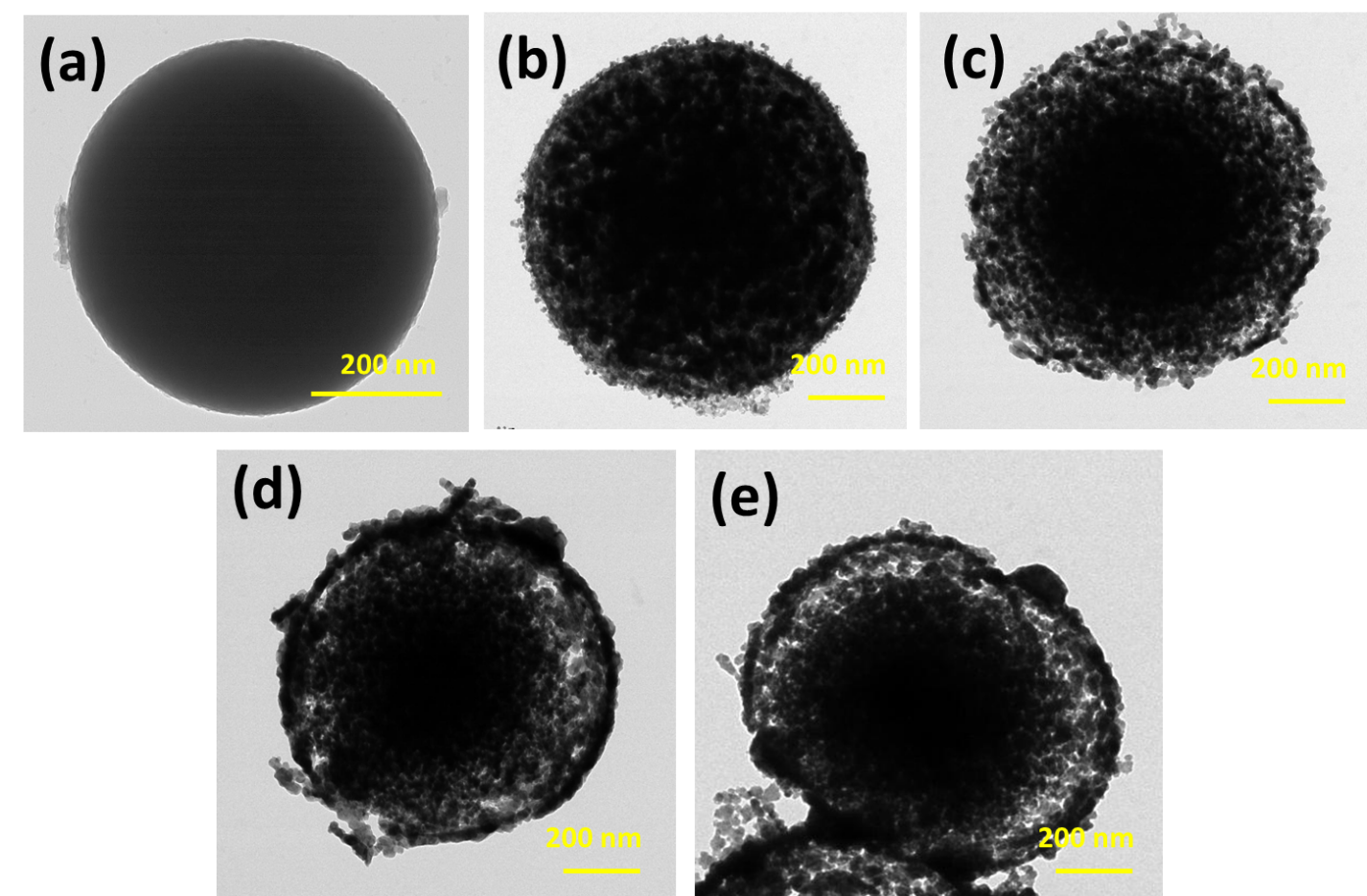


**Fig. S3** TEM images of the NiCoMn-glycerate concerning the sulfidation time (a) 0h, (b) 4h, (c) 6h, (d) 8h, and (e) 10h.


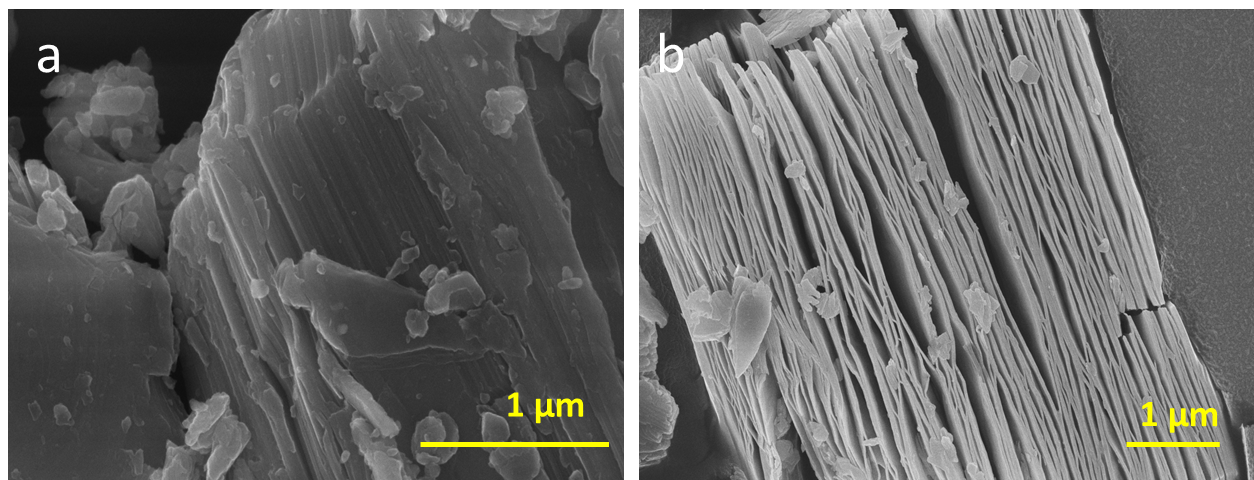


**Fig. S4** SEM image of the (a) MAX and (b) acid-etched MXene samples.

**
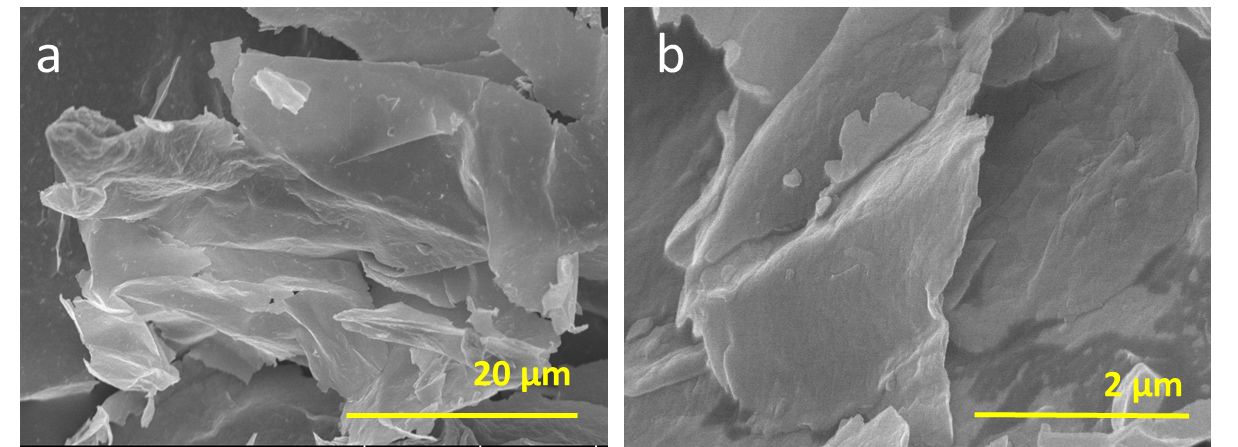
**

**Fig. S5** SEM image of the delaminated MXene samples.


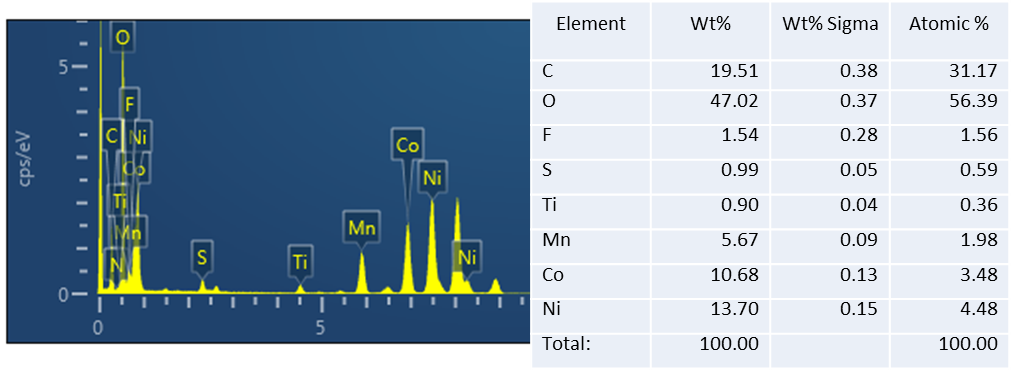


**Fig. S6** EDAX spectra of MXe-NiCoMn-LDH/S hollow spheres.


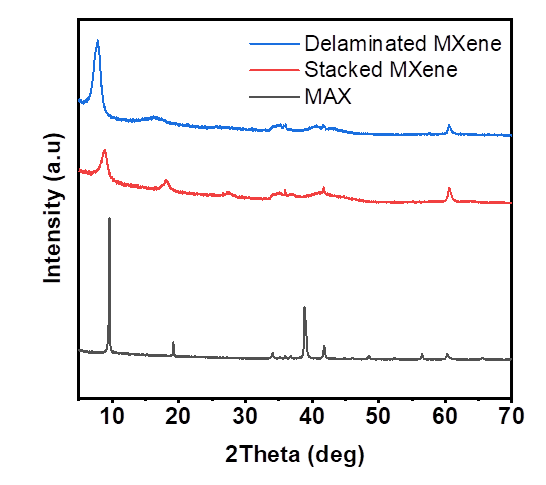


**Fig. S7** XRD spectra of the MAX, stacked MXene and delaminated MXene sheets.


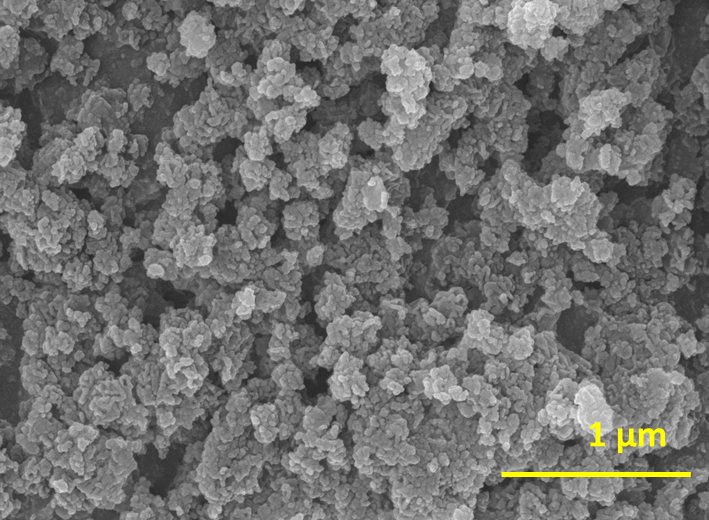


**Fig. S8**. SEM images of the MXe-NiCoMn-S samples prepared in the solvothermal process.


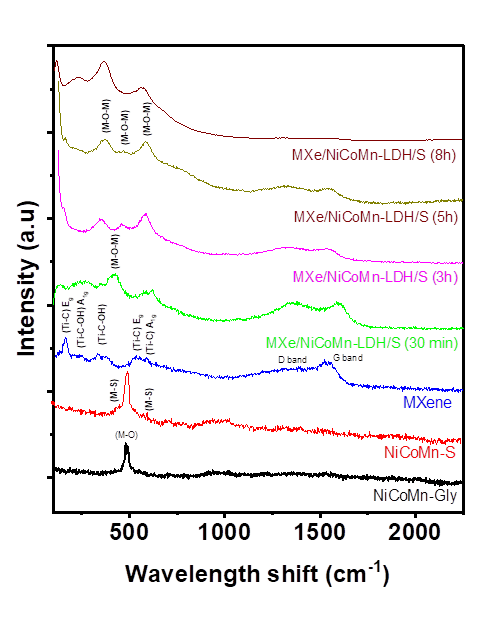


**Fig. S9** Raman spectra of the MXene, NiCoMn-Gly, and MXe-NiCoMn-LDH/S samples.


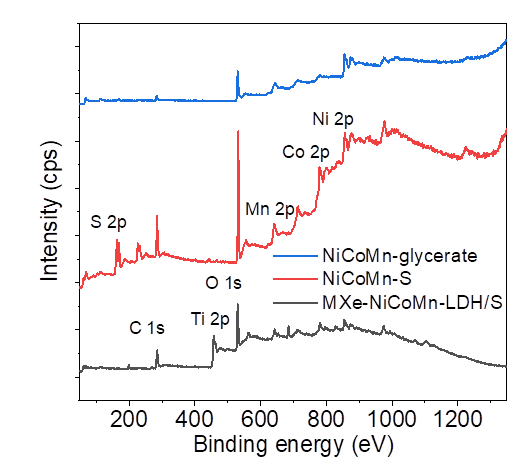


**Fig. S10** Survey spectra of the NiCoMn-glycerate, NiCoMN-S, and MXe/NiCoMn-LDH/S composite spheres.


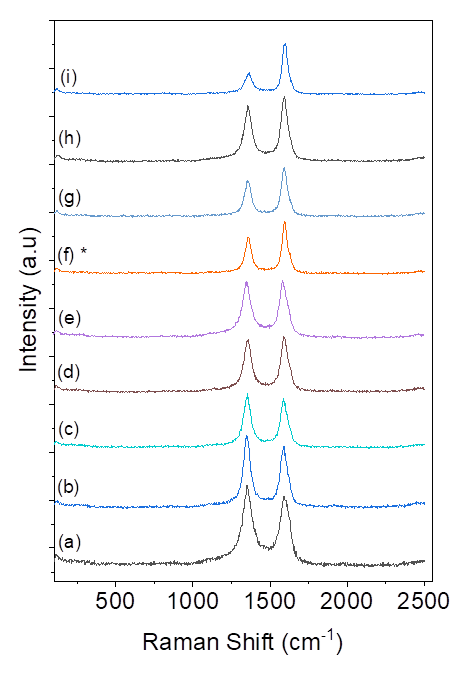


**Fig. S11** Raman spectra of the optimized LIG samples with different power and scanning speed.


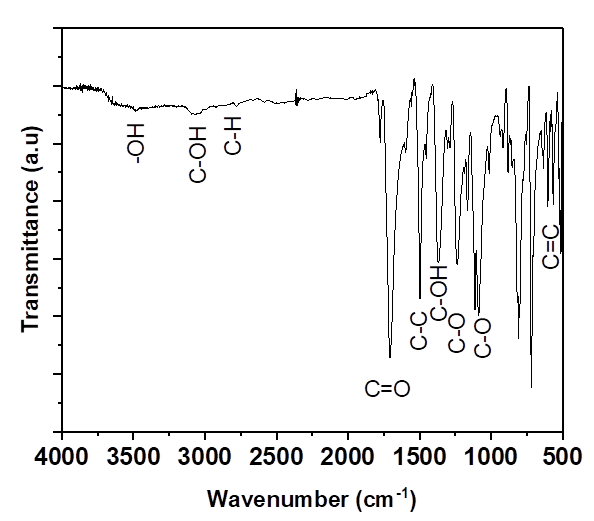


**Fig. S12** FTIR spectra of the optimized LIG substrate.

**
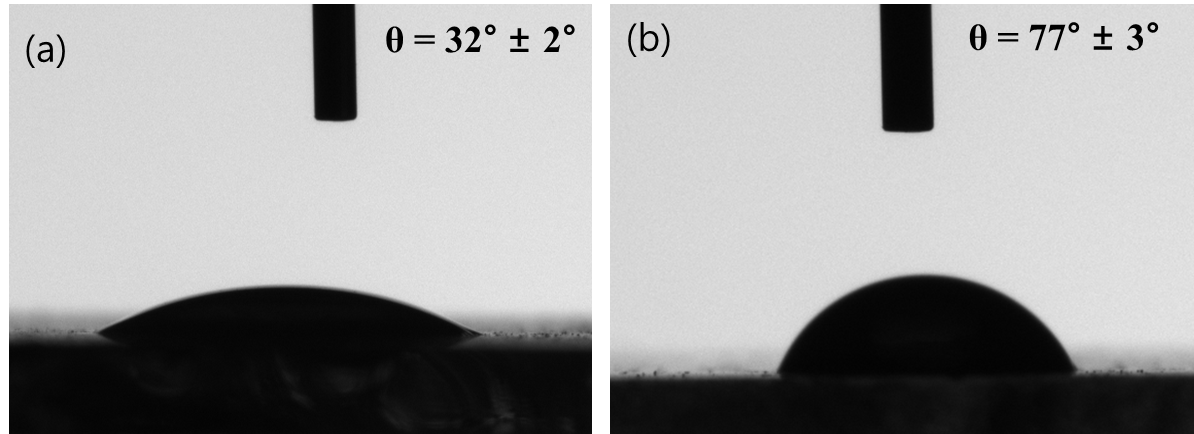
**

**Fig. S13** Water contact angle image of the (a) LIG and (b) GCD electrodes.

**
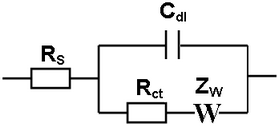
**

**Fig. S14**. Equivalent electrical circuit of EIS data for MXe-NiCoMn-LDH/S-LIG samples.

**
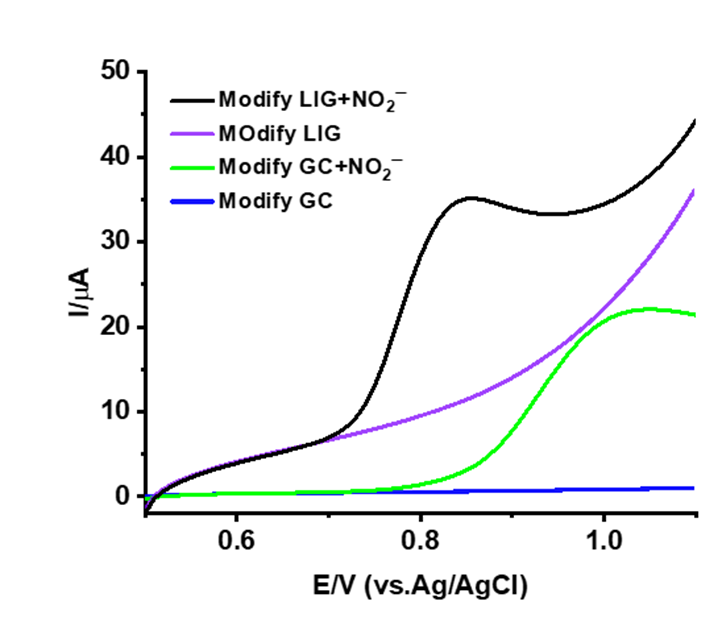
**

**Fig. S15** Comparative NO_2_- sensing performance of MXe-NiCoMn-LDH/S catalyst-loaded GC and LIG electrodes.


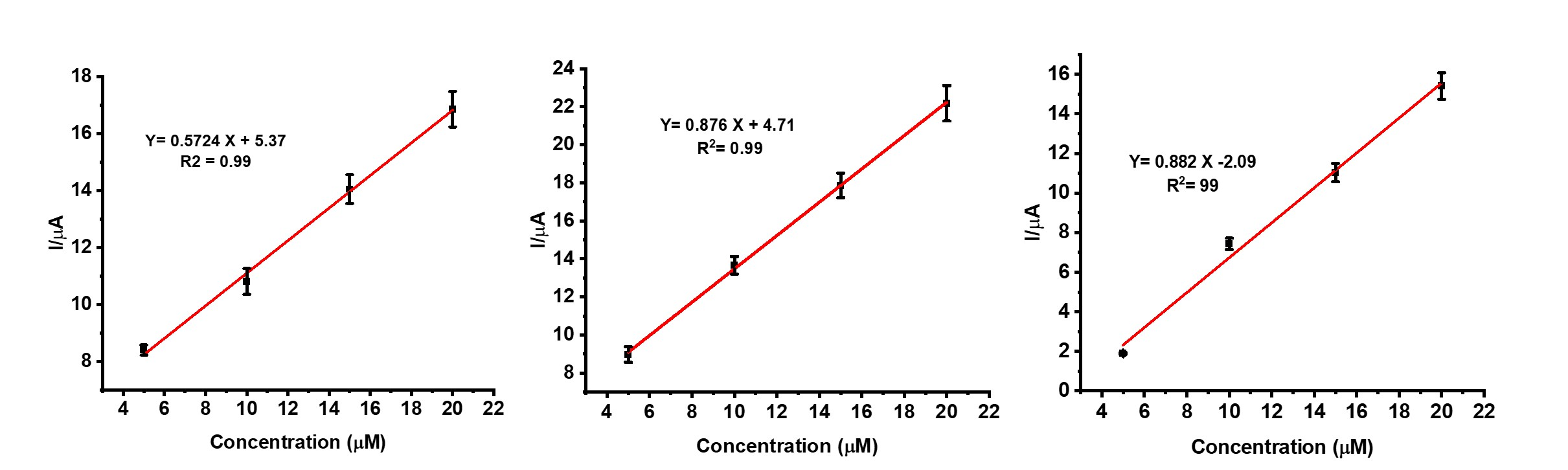


**Fig. S16** Calibration curves of real-time monitoring of NO_2_^-^ by the standard addition technique.

**Table S1.** Zeta potential data calculated for the NiCoMn-glycerate, NiCoMn-S, MXene, and MXe/NiCoMn-LDH/S composite sample under neutral pH

| **S. No** | **Sample** | **Zeta potential (mV)** |
| --- | --- | --- |
| 1 | NiCoMn-glycerate | 3.2 |
| 2 | NiCoMn-S | -4.2 |
| 3 | MXene | -21.2 |
| 4 | MXe/NiCoMn-LDH/S | 8.4 |

**Table S2.** ICP-OES data for the NiCoMn-glycerate, NiCoMn-S, and MXe/NiCoMn-LDH/S composite

| **Sample** | **Elemental Content (wt%)** | | | |
| --- | --- | --- | --- | --- |
|  | **Ni** | **Co** | **Mn** | **Ti** |
| NiCoMn-Glycerate | 27.23 | 15.37 | 16.48 | -- |
| NiCoMn-S | 22.36 | 21.87 | 0.87 | -- |
| MXe-NiCoMn-LDH/S | 13.45 | 11.74 | 7.24 | 1.32 |

**Table S3.** Surface composition of Ni, Co, Mn in the NiCoMn-glycerate, NiCoMn-S, and MXe/NiCoMn-LDH/S composite, which was measured through the XPS characterization

| **Sample** | **Atomic ratio (%)** | | | | | | |  |  |
| --- | --- | --- | --- | --- | --- | --- | --- | --- | --- |
|  | **Ni 2p** | **Co 2p** | **Mn 3d** | **Ti 2p** | **O 1s** | **C 1s** | **S 2p** | **F 1s** | **N 1s** |
| NiCoMn-Glycerate | 8.71 | 4.88 | 5.53 | -- | 44.56 | 36.32 | -- | -- | 1.87 |
| NiCoMn-S | 4.71 | 5.55 | 2.54 | -- | 36.27 | 30.49 | 19.32 | -- | 1.12 |
| MXe-NiCoMn-LDH/S | 6.14 | 5.04 | 4.74 | 4.52 | 42.06 | 23.48 | 10.30 | 2.14 | 1.58 |
